# Multi-Target Biological Activities of Podophyllotoxin-Derived Natural Products

**DOI:** 10.32604/or.2025.067666

**Published:** 2025-09-26

**Authors:** Yuhan Xie, Shaden A. M. Khalifa, Hesham R. El-Seedi, Paolo Coghi

**Affiliations:** 1 School of Pharmacy, Macau University of Science and Technology, Macau, 999078, China; 2 Neurology and Psychiatry Department, Capio Saint Göran’s Hospital, Sankt Göransplan 1, Stockholm, 112 19, Sweden; 3 Department of Chemistry, Faculty of Science, Islamic University of Madinah, Madinah, 42351, Saudi Arabia; 4 Key Laboratory of Quality Research in Chinese Medicine, Macau University of Science and Technology, Macau, 999078, China

**Keywords:** Podophyllotoxin, anticancer, structure-activity relationship, structural modification

## Abstract

Podophyllotoxin is a well-studied natural product. Because of its unique structure and ability to inhibit cancer cells, it has been changed in different ways to find out its pharmacological properties. This paper discusses the common chemical modifications of podophyllotoxin molecules, including the C-4 and E-4 site replacements. Furthermore, its common inhibitory effects on cancer cells and antiparasitic activities, among others, are outlined by the connection between conformational changes and pharmacological activities. Importantly, Podophyllotoxin can effectively overcome the phenomenon of multidrug resistance through a dual-targeting mechanism, including inhibition of microtubule protein synthesis and topoisomerase II activity, and induces cell cycle arrest and apoptosis. Recent findings reveal its potential to modulate immune responses through the cyclic GMP-AMP synthase (cGAS)-stimulator of interferon genes (STING) pathway, further extending beyond its classical mechanisms. This study finally provides a systematic summary of the activity of podophyllotoxin in common cancer cells, including those in the breast, lung, and prostate.

## 1 Introduction

**Figure 1:**
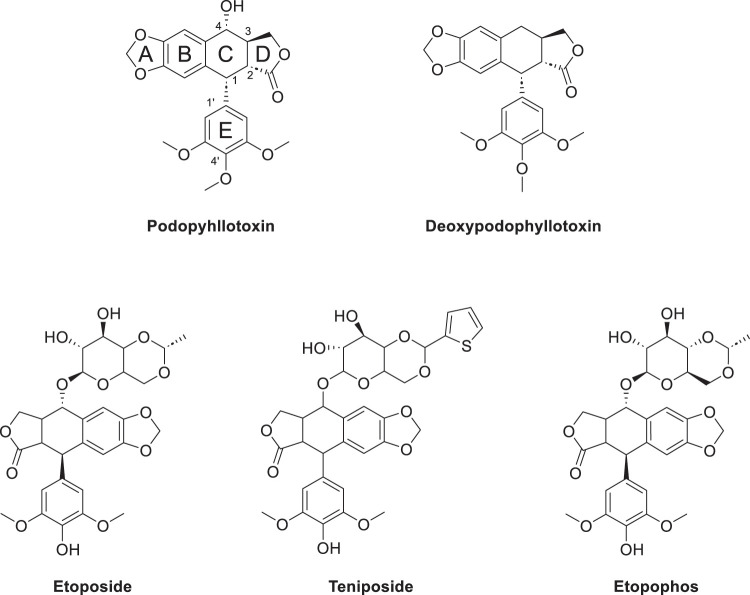
Podophyllotoxin (Consists of five rings (A–E)) and active derivatives. Created by ChemDraw (PerkinElmer, version 22.0, Waltham, MA, USA)

Podophyllotoxin (PTOX, Fig. 1) is a cyclolignan analog derived from plants of the genus Podophyllum, found primarily in the rhizomes of *Podophyllum peltatum* and *Podophyllum hexandrum* [1]. The Russian chemist Valerian Podwyssotzki first isolated the podophyllotoxin in 1880 [2], it was subsequently used in a wide variety of studies due to its unique stereochemical structure such as after binding with glucose derivatives, deoxypodophyllotoxin (Fig. 1) was found to form etoposide [3] (Fig. 1), binding to thiophene carboxylate produces teniposide (Fig. 1). Etopophos (Fig. 1), the phosphate prodrug of Etoposide. Etoposide causes DNA double-strand breaks by inhibiting the activity of DNA topoisomerase II, preventing the cell from entering mitosis and stalling in G2/M phase. Etopophos enhances water solubility through phosphate ester structure and is commonly used for intravenous injection. Podophyllotoxin derivatives play a crucial role in overcoming several challenges in modern cancer therapy, such as multidrug resistance and nonspecific toxicity. By precisely targeting DNA topoisomerase II and inducing controlled DNA damage, these derivatives have the potential to increase efficacy against rapidly proliferating tumor cells while minimizing damage to normal tissues [4].

**Figure 2:**
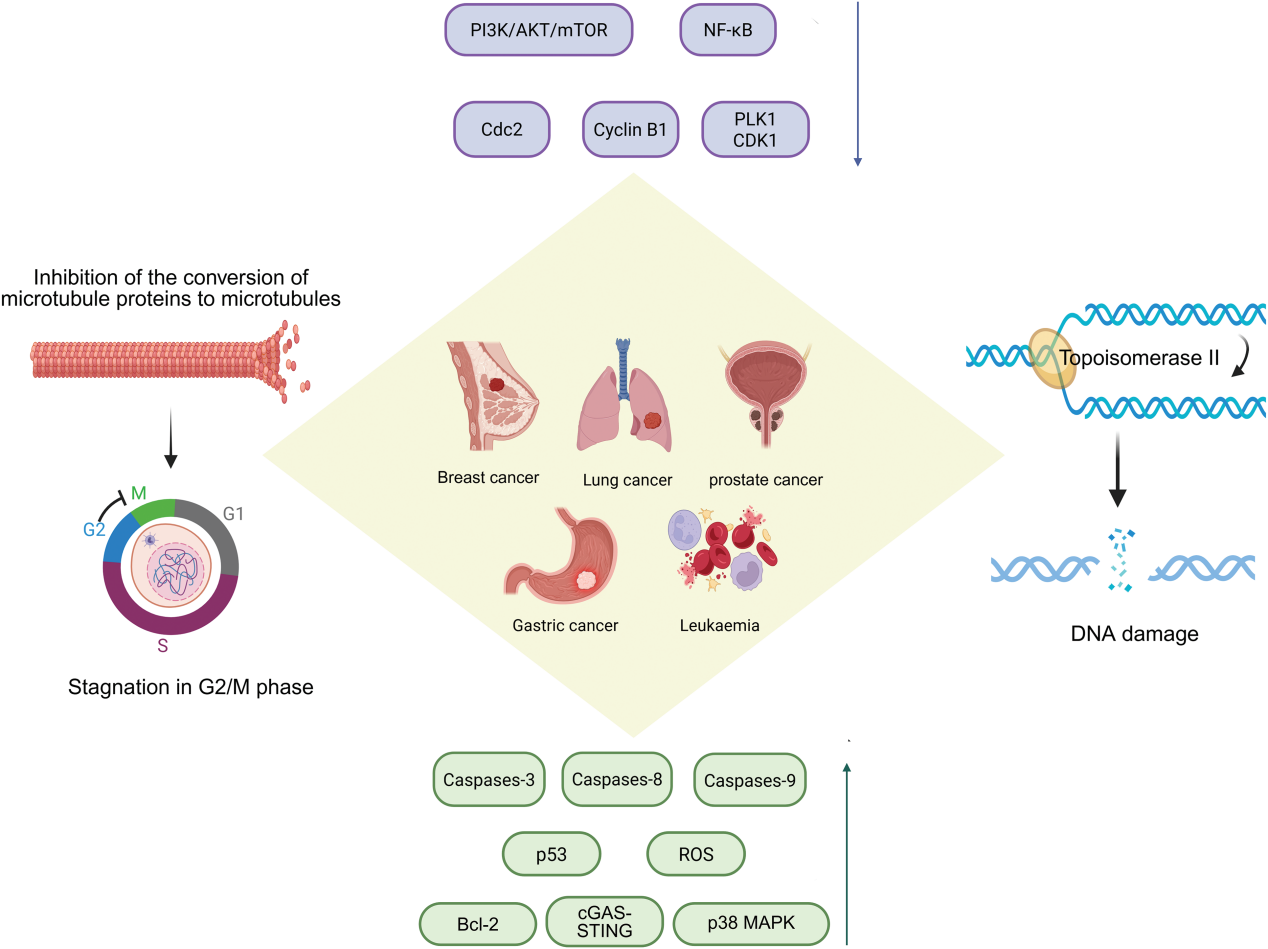
Podophyllotoxin acts by inhibiting microtubule protein synthesis, topoisomerase II activity, and regulating various signaling pathways. Podophyllotoxin has dual anticancer mechanisms: the inhibition of microtubule assembly disrupts mitotic spindle formation, leading to G2/M cell cycle arrest; inhibition of topoisomerase II induces DNA double-strand breaks, resulting in genomic instability and cell death. These effects activate key signaling pathways involved in cell proliferation and survival, collectively contributing to the therapeutic effects against various cancers, including breast, lung, prostate, gastric cancer, and leukemia. PI3K/Akt/mTOR, Phosphoinositide 3-kinase/Protein kinase B/Mechanistic Target of Rapamycin; NF-κB, Nuclear Factor κB; Cdc2, Cell Division Cycle 2; PLK1, Polo-Like Kinase 1; CDK1, Cyclin-Dependent Kinase 1; ROS, Reactive Oxygen Species; cGAS-STING, cyclic GMP-AMP synthase-stimulator of interferon genes; p38 MAPK, p38 Mitogen-Activated Protein Kinase. Created in https://BioRender.com

Unlike the mechanism by which paclitaxel analogues block microtubule depolymerization, podopyllotoxin binds to the hydrophobic pocket of the β-tubulin subunit, inhibiting the conversion of microtubulin to microtubules and disrupting the dynamic equilibrium between microtubule tubules and microtubulin, thereby inhibiting the process of mitosis and arresting the cell cycle in G2/M phase. It is evident that the colchicine binding site is also present here [5]. Podophyllotoxin not only binds to the target site more rapidly, but also a reversible process [6]. Podophyllotoxin derivatives inhibit PI3K/Akt/mTOR and NF-κB signalling pathways, reduce inflammatory response signals and induce apoptosis and autophagy, activate the p38 MAPK pathway and arrest the cell cycle by increasing reactive oxygen species production [7]. Not only that, studies have shown that podophyllotoxin can also concentration-dependently inhibit apoptosis in certain oxidative stress or non-tumour cell environments [8]. It is cytoprotective by up-regulating the expression of Bcl-2 (anti-apoptotic protein), maintaining the integrity of mitochondria, inhibiting the activity of caspase-3, and blocking the process of DNA breakage (Fig. 2).

However, PTOX has unavoidable toxicity problems in the clinical treatment, mainly manifested as gastrointestinal reactions, liver impairment and bone marrow suppression, which limits the progress of treatment, affects patient tolerance and leads to a decrease in therapeutic efficacy, and the use of nanodelivery systems or premedication strategies is considered to reduce the exposure, decrease the toxicity and improve the bioavailability. Researchers apply the podophyllotoxin backbone to the synthesis of protein degradation targeting chimeras [9], ligating an E3 ubiquitin ligase ligand in derivatives of podophyllotoxin specifically degrades the relevant proteins and overcomes the drug resistance problem. In terms of immunity, podophyllotoxin disrupts the cellular microtubule structure, alters the intracellular transport of the STING signalling pathway, enhances the strength of the cGAS-STING signalling pathway (Fig. 2), and produces more immune factors to inhibit tumour growth [10,11].

Despite the continuous progress and improvement in the treatment of cancer, cancer is still one of the most difficult problems in the world due to its difficult treatment, easy recurrence, and different physical conditions of patients. According to the World Health Organization, cancer is the second leading cause of death worldwide, followed first one which is cardiovascular diseases [12]. The most important reason for this is that cancer cells mutate at a very high frequency, making well-activated compounds no longer effective. This specific resistance phenomenon is caused by mechanisms such as overactive efflux pumps, enhanced DNA repair, reduced drug uptake, and altered target sites [13]. Therefore, products with multi-target anticancer mechanisms and their derivatives have been valued, as they can effectively overcome the resistance phenomenon that accompanies a single target. Structural modification studies around the podophyllotoxin skeleton are expected to provide more drug candidates for clinical use and address the problem of multidrug resistance.

## 2 Conformational Relationships

**Figure 3:**
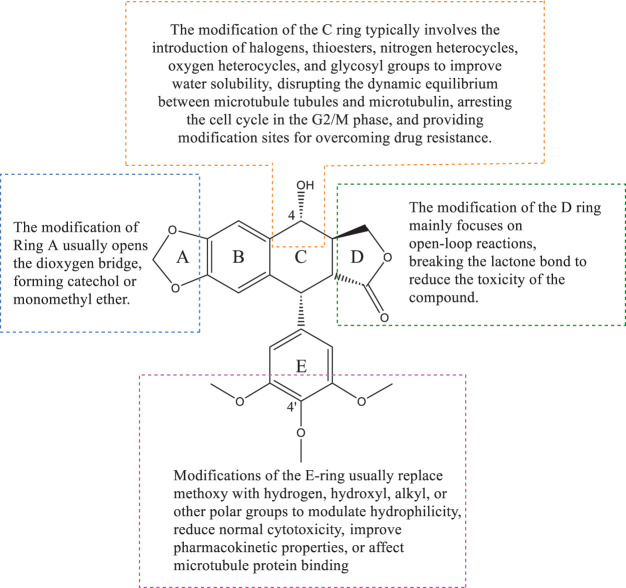
This figure shows the overall idea of structure modification, derivative design, and pharmacological activity study of podophyllotoxin as the core; different modification sites will have different biological activity changes. Created by ChemDraw (PerkinElmer, version 22.0)

Podophyllotoxin is a polycyclic compound, consisting of four stereogenic centers and five rings (A–E) (Fig. 3), of which the A–D rings form a rigid tetracyclic core and the E ring is aromatic; modification of either part can affect its activity (Table 1) [11,14,15]. Most of the hybrids are superior to current clinical drugs in terms of inhibitory effect and selectivity, offering more promising derivatives to address drug resistance and improve therapeutic efficacy [16]. Modifications are often centered on the C-4 of the C-ring and the C-4^′^ site of the E-ring, enhancing anticancer activity through the introduction of C-C, C-N, C-O and C-S bonds [17], the alteration of the spatial resistance at C-4 results in a variety of biologically significant pharmacologically relevant activities [18,19]. For example, the incorporation of an amino group at the C-4 position enhances the affinity for binding to microtubules, thereby increasing anti-mitotic activity [20]. The D-ring modification has the capacity to reduce toxicity by opening the lactone bond.

**Table 1:** Common modification sites of podophyllotoxin, structure-activity relationship, and biological activity

C-4 modified derivatives						
**Compound**	**R-Group**	**A-549**	**PC-3**	**MCF-7**	**Sar**	**Ref**.
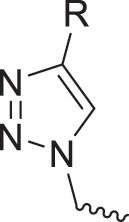	R = CH_2_OH	>100 μM	0.06 μM	0.01 μM	Continued extension of the carbon chain length decreases the activity; too long carbon chains affect the binding rate to the target and different cell lines have different sensitivities to the side chain length.	[30]
	R = Ethyl	35 μM	0.03 μM	0.4 μM		
	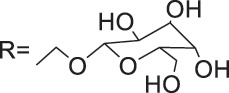	4.07 μM	/	7.28 μM	/	[31]
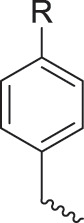	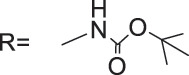	0.0249 ± 0.0044 μM	0.0158 ± 0.0019 μM	0.0345 ± 0.0077 μM	Aromatic substitutions were superior to aliphatic groups, especially the substituents containing electron donors, to improve the affinity of the compounds to the target proteins.	[32]
	R = F	0.0359 ± 0.0091 μM	0.06 ± 0.0087 μM	0.0434 ± 0.0051 μM		
	R = COOMe	0.0301 ± 0.0062 μM	0.0211 ± 0.0011 μM	0.0252 ± 0.0033 μM		
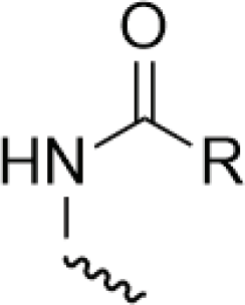	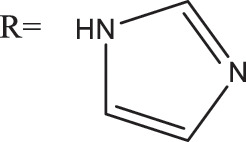	/	/	25 ± 2 μM	Substituted 4β-N-substituted compounds containing amino groups were overall more potent than 4β-O-substituted and less toxic to normal cells.	[33]
	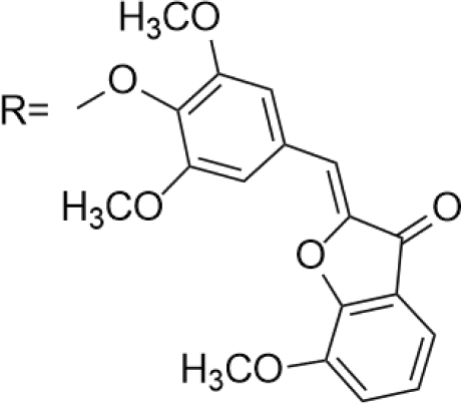	1.45 ± 0.77 μM	/	0.23 ± 0.081 μM	H substituents in the benzene and furan rings showed positive activities. When the substituents were all replaced with methoxy, the inhibitory effect was slightly reduced, but still better than etoposide.	[34]
	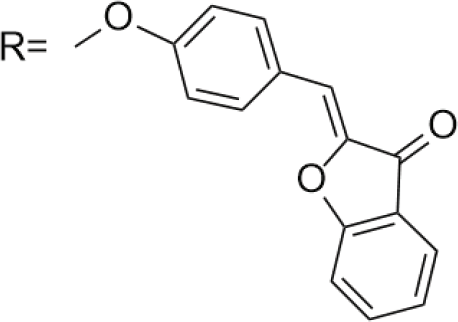	0.10 ± 0.072 μM	/	0.13 ± 0.087 μM		
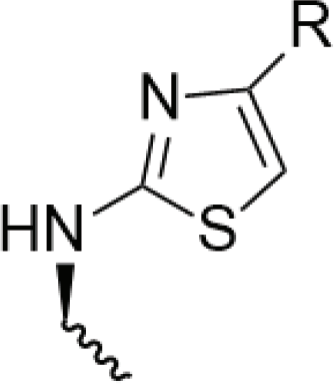	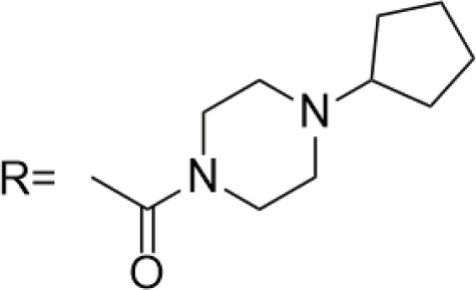	1.3 ± 0. 9 μM	/	/	The results showed that the derivatives with amine groups at the end were unstable in activity, and the change to ester substitutions resulted in enhanced hydrophobicity and lower toxicity.	[35]
	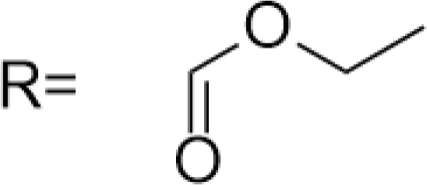	0.16 ± 0.06 μM	/	/		
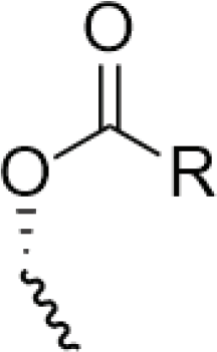	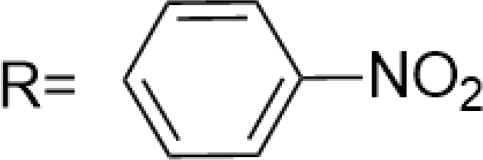	0.74 ± 0.28 μM	/	1.56 ± 0.69 μM	Containing alkynoate derivatives were the most active, suggesting that the terminal alkynyl group can influence the action on microtubule targets, and acrylates were moderately active, but the introduction of too many aromatic rings reduced the activity.	[36]
	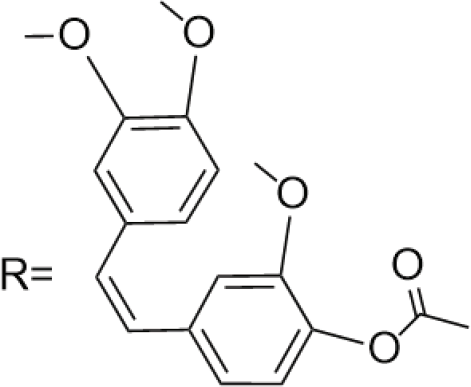	0.91 ± 0.497 μM	/	1.83 ± 0.94 μM		
	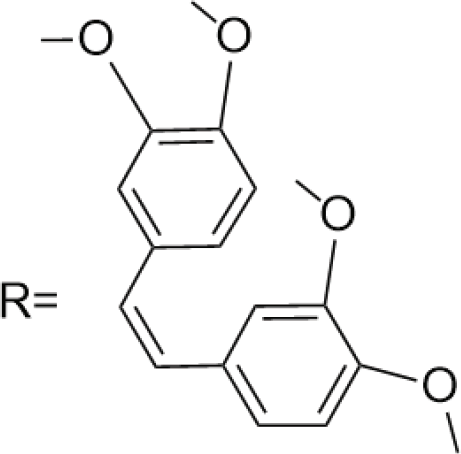	0.71 ± 0.38 μM	/	1.74 ± 0.608 μM		
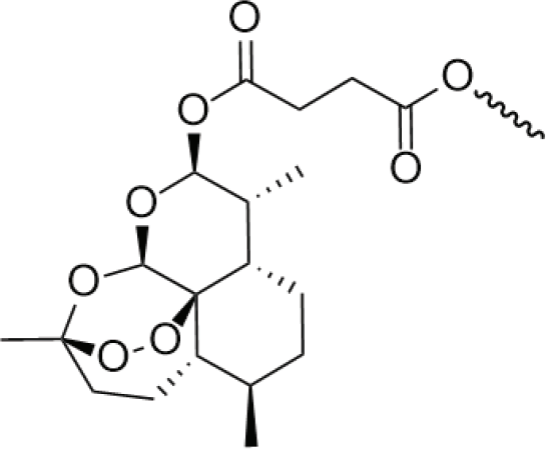		3.011 ± 0.272 μM	/	/	The combination of the podophyllotoxin and ART has excellent combined activity and mitigating effect on drug resistance, which has good potential for development	[37]
**E-4 Modified Derivatives**						
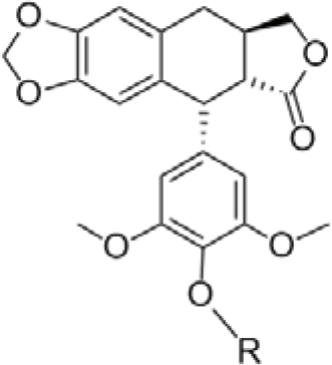	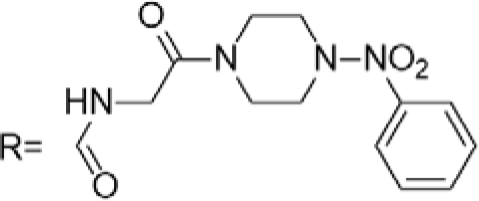	0.102 μM	/	/	The presence of the p-nitrophenyl piperazine moiety enhances hydrophobic and electronic interactions. Further improvement is achieved by introducing L-amino acids, providing favorable stereochemistry and additionalπ-π interactions.	[38]
	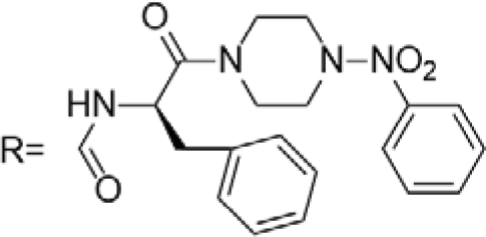	0.0802 μM				
	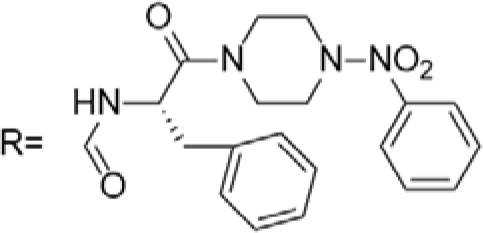	0.300 μM				
	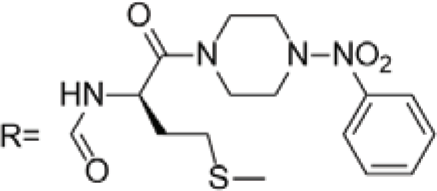	0.183 μM				
	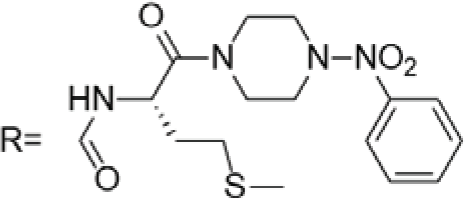	0.208 μM				

Note: SAR, structure-activity relationship.

Deoxypodophyllotoxin (DPT) is a key derivative of podophyllotoxin, obtained by removing the hydroxyl group at the C-4 position, which enhances cell membrane permeability and metabolic stability [21,22]. DPT can regulate a variety of signaling molecular pathways and cellular life processes, and exhibits good activity against a variety of tumor cells, inducing programmed cell death by activating the mitochondrial pathway, inhibiting the expression of CDK1 to cause cells to stagnate in the G2/M phase, and at the same time increasing the level of ROS to promote apoptosis [23]. It is worth investigating that DPT can induce necroptosis and parthanatos, two different mechanisms of programmed cell death pathways distinct from apoptosis, both of which act independently of caspases and can be activated simultaneously in the same cell (Fig. 3). Necroptosis is a form of programmed necrotic cell death mediated by the RIPK1-RIPK3-MLKL signaling axis, which is activated primarily in the absence of caspase-8, triggering cytolysis and the release of damage-associated molecular patterns (DAMPs), ultimately leading to cellular necrosis [24–26]. Parthanatos is also a way to induce cell death without activating caspases, caused by overactivation of PARP-1 [27], which causes the release of AIF from the mitochondria, which triggers DNA breaks and ultimately leads to cell death [28,29].

## 3 Biological Activity

Podophyllotoxin and its derivatives have been structurally modified to produce a variety of types with different anticancer mechanisms, such as interfering with microtubule polymerization, inhibiting topoisomerase II activity, inducing apoptosis, antiangiogenesis, and antiproliferation, etc., which can act singly or simultaneously in cells, conferring podophyllotoxin with unique anticancer activities [39–41].

Recent clinical trials have brought renewed attention to podophyllotoxin and its structurally modified derivatives as potent candidates in anticancer drug development. Based on the extensively characterized cytotoxic properties of this natural lignan, researchers have focused on optimizing its efficacy, selectivity, and pharmacokinetic properties through strategic chemical modifications. Notably, derivatives featuring improved solubility, reduced systemic toxicity, and enhanced tumor-targeting capabilities are now progressing through various stages of clinical evaluation. This section highlights key findings from these recent studies and explores the therapeutic potential and challenges that lie ahead for podophyllotoxin-based agents in modern oncology (Table 2).

**Table 2:** Some podophyllotoxin-derived clinical trials

Clinical trials ID	Description	Department	Status	Year	Ref.
NCT01492556	A Phase II, Single-arm, Multicenter Study of Etoposide Monotherapy in Treating Patients with Recurrent or Metastatic Breast Cancer	Chinese Academy of Medical Sciences	Unknown Status	2011–2015	[42]
NCT03923179	The Efficacy and Safety of Pyrotinib Combined with Etoposide in HER2-positive Advanced Breast Cancer Conditions	Cancer Institute and Hospital, Chinese Academy of Medical Sciences	Unknown Status	2019–2021	[43]
JCOG 9702	Randomized phase III trial of carboplatin plus etoposide vs. split doses of cisplatin plus etoposide in elderly or poor-risk patients with extensive disease small-cell lung cancer	Japan Clinical Oncology Group (JCOG), National Cancer Center, Tokyo, Japan	Completed	1998–2004	[44]
220038	Carboplatin in combination with etoposide for advanced small cell lung cancer complicated with idiopathic interstitial pneumonia: a single-arm phase II study	Nippon Medical School Hospital	Completed	2009–2022	[45]
NCT01642251	Cisplatin and Etoposide with or Without Veliparib in Treating Patients with Extensive Stage Small Cell Lung Cancer	National Cancer Institute	Completed	2012–2018	[46]
NCT04878016	A Phase III, Randomized, Double-blind Placebo-controlled Study of Carboplatin Plus Etoposide with or without ZKAB001 (Anti-PD-L1 Antibody) in Patients With Untreated Extensive-stage Small Cell Lung Cancer	Lee’s Pharmaceutical Limited, Hong Kong, China	Completed	2021–2023	[47]
ChiCTR2300074591	Prospective study of neoadjuvant adabrelimab combined with chemotherapy in limited-stage small cell lung cancer	Lung Cancer Center, West China Hospital, Sichuan University	Active	2023	[48]
NCT03043872	A Phase III, Randomized, Multicenter, Open-Label, Comparative Study to Determine the Efficacy of Durvalumab or Durvalumab and Tremelimumab in Combination with Platinum-Based Chemotherapy for the First-Line Treatment in Patients with Extensive Disease Small-Cell Lung Cancer (SCLC) (CASPIAN)	Department of Medical Oncology, Hospital Universitario 12 de Octubre, Madrid, Spain	Active not recruiting	2017–2018	[49]
NCT00878449	A Phase 1 Study Evaluating the Safety of ABT-263 in Combination with Etoposide/Cisplatin in Subjects with Small Cell Lung Cancer (SCLC)	AbbVie, USA	Completed	2009–2011	[50]
Not applicable	Randomized phase III clinical trial comparing gemcitabine/cisplatin and etoposide/cisplatin in non-small cell lung cancer	Multicenter study (Spain); Coordinated by Hospital Germans Trias i Pujol and supported by Eli Lilly S.A.	Completed	1996–1997	[51]
NCT02773732	A Phase Ib/II Clinical Trial of Oral Ciprofloxacin and Etoposide in Subjects with Resistant Acute Myeloid Leukemia (AML)	Department of Medicine, University of Florida, Gainesville, FL, USA	Terminated	2016–2021	[52]
NCT01457040	A Prospective Study of Intensified Conditioning Regimen with High-Dose Etoposide for Allogeneic Hematopoietic Stem Cell Transplantation for Adult Acute Lymphoblastic Leukemia in China	Nanfang Hospital, Southern Medical University	Completed	2011–2016	[53]
Not applicable	Clinical trial of etoposide and cisplatin as salvage therapy in advanced ovarian carcinoma	Division of Gynecologic Oncology, The Mount Sinai Medical Center, The Mount Sinai School of Medicine, New York, NY, USA	Completed	1984–1986	[54]
NCT03963193	Phase II Trial of Etoposide Plus Cisplatin Compared with Irinotecan Plus Cisplatin for First-line Treatment of Non-primary Pancreatic Metastatic and/or Unresectable Gastrointestinal Neuroendocrine Tumor G3 Type	Henan Cancer Hospital, Zhengzhou, China	Unknown Status	2019–2021	[55]
NCT00513162	Valproate (Valproic Acid) and Etoposide for Patients with Progressive, Relapsed or Refractory Neuronal Tumors and Brain Metastases	M.D. Anderson Cancer Center, Houston, TX, USA	Completed	2007–2012	[56]
NCT04154189	A Multicenter, Open-label, Randomized Phase 2 Study to Compare the Efficacy and Safety of Lenvatinib in Combination with Ifosfamide and Etoposide vs. Ifosfamide and Etoposide in Children, Adolescents and Young Adults with Relapsed or Refractory Osteosarcoma (OLIE)	Eisai Inc., Merck Sharp & Dohme LLC.	Completed	2020–2023	[57]

### 3.1 Breast Cancer

Breast cancer is one of the most common types of malignant tumors in women worldwide [58–60], affecting a wide range of women and seriously endangering their health, and despite advances in modern treatments, drug resistance remains a major problem. Deep research into the drug resistance mechanism of breast cancer and the search for novel targeted drugs are the main directions of current research and treatment. Podophyllotoxin can significantly inhibit the growth and spread of cancer cells by blocking the normal cell cycle through inhibiting PLK1/CDK1 and suppressing the expression of EMT-related proteins (e.g., N-cadherin, MMPs), but its development has been limited by high toxicity and other drawbacks. For this reason, a series of structurally modified derivatives have been derived, such as the introduction of triazole rings, amide, alkane or ether bonds to improve selectivity and minimize toxic side effects [61].

**Figure 4:**
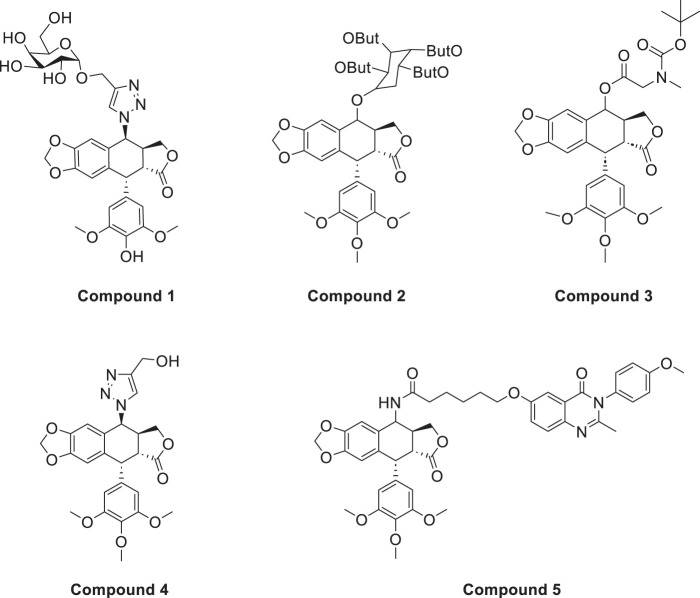
Excellent anticancer activity of podophyllotoxin derivatives 1–5 in breast cancer. Created by ChemDraw (PerkinElmer, version 22.0)

Azoles such as 1,2,3-triazole, tetrazole, thiazole, and imidazole are a class of structures widely used in anticancer drug design, which not only improve targeting to enhance activity [62,63], but also form a variety of derivatives through click chemistry, providing a wide range of candidate compounds for research. Zi et al. [64] introduced a 1,2,3-triazole ring at the C-4 site of podophyllotoxin and attached the triazole ring to different sugar moieties by click chemistry. Among them, compound 1 (Fig. 4) exhibits good anticancer activity. 4β-triazole-podophyllotoxin glycosides exhibit selective inhibition of *in vitro* anticancer activity in breast cancer MCF-7 cells [31] and display lower cytotoxicity. Moreover, stability investigation revealed that the incorporation of the galactose moiety slows down the hydrolysis and improves the chemical stability of the podophyllotoxin scaffold. Therefore, comparison of the measurements with etoposide and cisplatin revealed that the after modification derivatives showed significant activity with IC_50_ = 7.28 μM as compared to 32.82 and 15.86 μM for etoposide and cisplatin.

In the process of natural product anticancer drug development, several studies have shown that the introduction of glycosidic structures and amino acids can improve the anticancer activity of drugs [65,66]. Introduction of an ester group or amide bond at the C-4 position also enhances the activity, 4β-N-substituted was active against a wide range of cancer cells, considering that the nitrogenous heterocycle enhances topoisomerase II inhibition, resulting in an increase in cell permeability, and the imidazole substitution also results in a strong aqueous solubility and improves bioavailability.

Zi et al. [67,68] introduced glycosidic moieties into the C-4 position of three different podophyllotoxin-like skeletons, affording a series of novel fully butyrylated glycosidylated podophyllotoxin derivatives, among which compound 2 (Fig. 4) was the most active to MCF-7 and SW480 cells. The different glycosidic substituents also had a great influence on the anticancer activity, D-galactose and D-arabinose were significantly better than glucose and mannose. The per-butyrylated glycosides of podophyllotoxin exhibited superior inhibitory activity to Etoposide against MCF-7. The glycosidic structure may increase the efficiency of cellular uptake, and the butyrate moiety possesses HDAC inhibitory activity, induces apoptosis, and enhances selective toxicity.

Wu’s team attached different Boc-protected amino acids to podophyllotoxin and then used the deprotection technique to obtain a new batch of amino acid derivatives, compound 3 (Fig. 4), with an IC_50_ of 7.2 nM on MCF-7 cells, which is hundreds of times stronger than etoposide [69]. The Boc protection improved the selectivity compared to the deprotected derivatives, but the activity was not as high as that of the Boc-protected derivatives, providing guidance for further modifications of podophyllotoxin.

Another class of similarly structured podophyllotoxin derivatives also showed enhanced activity compared to the clinical therapeutic drug etoposide [30]. Staining results showed that triazole derivatives induced significant MCF-7 cell apoptosis, and that the attachment of short-chain alkanes and hydroxyl groups to the triazole ring increased the anticancer activitysuch as compound 4 (Fig. 4), but continued extension of the carbon chain length decreases the activity, considering that the hydrophobicity of too long carbon chains affects the binding rate to the target and that different cell lines have different sensitivities to the side chain length, providing a research direction for finding potential targets of action.

Kamal’s research team synthesized a series of novel quinazoline-podophyllotoxin conjugates [70]. It was found to upregulate the expression of p53 protein and cyclin B1 protein, thereby controlling the transition from G2 to M phase and inhibiting mitosis. The conjugates not only retain the core structure responsible for inhibiting microtubule formation but also interfere with signaling pathways, thereby blocking angiogenesis. Moreover, its mechanism involves inducing mitochondrial damage, increased caspase-9 levels, and apoptosis. Compound 5 (Fig. 4) was the most active, and the change to halogen substitution resulted in slightly lower activity than compound 5.

Comparing DPT with isocupressic acid, an extract of Juniperus communis, it was found that DPT enhanced IκBα expression and thus inhibited NF-κB transcriptional activity, and the inhibition process could bypass the p53 pathway, which showed a good inhibitory activity against breast cancer [71].

In breast cancer cells, topoisomerase II is an important therapeutic target. However, the high genetic heterogeneity of breast cancer and the poor response of some subtypes limit the generalization of this class of drugs. In the future, it can be combined with multi-target action as well as individualized therapy to achieve better clinical efficacy [34].

### 3.2 Lung Cancer

**Figure 5:**
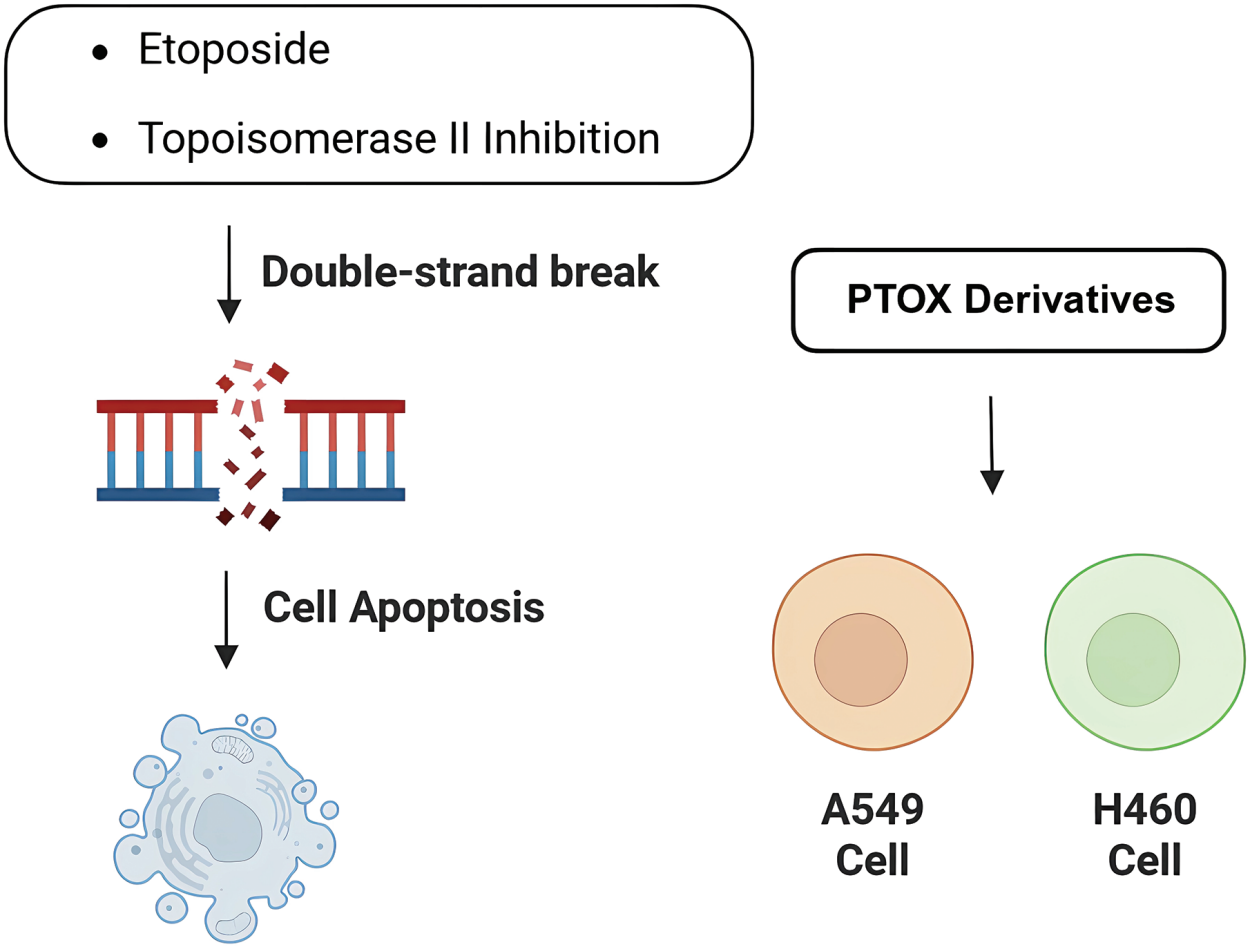
Activity of podophyllotoxin and its derivatives in lung cancer. Podophyllotoxin and the derivative Etoposide cause DNA double-strand breaks and induce apoptosis by inhibiting topoisomerase II activity. Also exhibited potential antitumor activity against different lung cancer tumor cell lines (e.g., A549 and H460 cells). Created in https://BioRender.com

Lung cancer has two categories: non-small cell lung cancer (NSCLC) and small cell lung cancer (SCLC), is the second most common cancer [72], and the incidence of which is closely related to a variety of genetic factors and the environment, and this type of disease has threatened the normal life activities of human beings. Currently, in addition to traditional immunotherapy and combination chemotherapy of etoposide and platinum, the treatment of lung cancer has been gradually carried out with precision medicine therapy, and common targets include EGFR, ROS1, KRAS, etc. The structure of podophyllotoxin has been extensively modified, and the already marketed derivative Etoposide inhibits Topoisomerase II, causing DNA strand breaks and subsequent apoptosis (Fig. 5). Recent single-molecule studies have further revealed that Etoposide not only promotes the capture and stabilization of DNA loops by Topoisomerase II, but also causes DNA to form a locked structure between the DNA-gate of Topoisomerase II and the closed C-gate, which severely alters the three-dimensional structure and function of chromatin [73]. This mechanism is highly dependent on ATP hydrolysis, suggesting that Topoisomerase II is required to perform the energy-driven cross-strand translocation step for drug action. In addition, Etoposide can significantly increase the stability and breaking force of DNA breakage complexes, causing Topoisomerase II to be fixed on DNA, hindering DNA replication, transcription and superhelical processes. It further exacerbates genomic instability, ultimately leading to strong cytotoxic effects. This multi-layered inhibitory mechanism gives Etoposide the unique advantage of efficiently inducing apoptosis in tumor chemotherapy. However, because of drug resistance problems, combinations of drugs have to be used, and the development of new derivatives has become a major task nowadays. Podophyllotoxin and its derivatives have shown good activity in human non-small cell lung cancer cell lines (e.g., A549, H460) [74].

**Figure 6:**
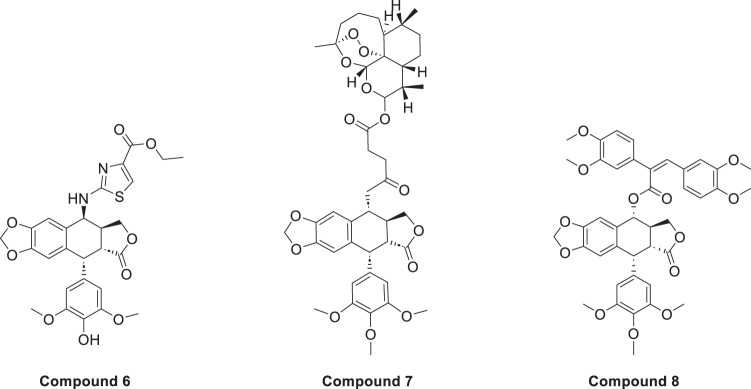
Excellent anticancer activity of podophyllotoxin derivatives 6–8 in lung cancer. Created by ChemDraw (PerkinElmer, version 22.0)

Sang’s team [35] found the optimal structure by varying the substituents at the end of the side chain. Derivatives with amine groups at the end were unstable in activity, but their structures were easy to modify. The change to ester substitutions in compound 6 (Fig. 6) resulted in enhanced hydrophobicity and lower toxicity. This modification method of introducing an amide bond at the C-4β position also shows good potential in fighting lung cancer [35]. In A549 cells, it can induce enhanced expression of p-ATM and p73 pathway and induce DNA damage response. Meanwhile, using WI-38 as a reference control, it was found that the derivatives were less toxic to WI-38, indicating better selectivity. In addition, the derivative induced DNA double-strand breaks through dose-dependent inhibition of topo-II, a mechanism of action similar to that of etoposide. The structural modification method of introducing an amide bond at the C-4β position also shows good potential in fighting lung cancer. They focused on a series of novel 4β-(thiazol-2-yl)amino-4^′^-O-demethyl-4-deoxypodophyllotoxins and explored their mechanisms of action in detail. These compounds act primarily by inhibiting topoisomerase II, an enzyme responsible for managing DNA supercoiling during replication. By stabilizing the DNA-topo II cleavable complex, they interfere with DNA religation and induce double-strand breaks (DSBs), ultimately triggering apoptosis in cancer cells. In addition, the compounds activate the p73/ATM pathway, which plays a central role in the cellular response to DNA damage. This leads to phosphorylation of H2AX (γ-H2AX), a key marker of DSBs, further amplifying the DNA damage signal and promoting cell cycle arrest. Together, topoisomerase II inhibition and activation of the p73/ATM/γ-H2AX axis contribute to the enhanced anticancer activity observed in the tested compounds. *In vitro* experiments using the A549 human lung carcinoma cell line revealed that these derivatives significantly suppress tumor cell proliferation. The compounds were tested through a standard 48 h MTT assay, where cells were treated with different concentrations of the synthesized molecules, and IC_50_ values were determined. Furthermore, cytotoxicity was evaluated in normal human lung fibroblasts WI-38, showing that the compounds exhibited much lower toxicity in normal cells, highlighting their therapeutic potential and improved safety profile. DPMA exhibited superior antiproliferative activity to etoposide on A549 cells and lower toxicity to normal human-derived mesenchymal stem cells (MSCs).

Artemisinin is widely used for its antimalarial activity, and its esterification to give artesunate (ART). Coupling of artesunate with podophyllotoxin via esterification reaction gave compound 7 (Fig. 6) [37]. The *in vitro* antiproliferative results showed that podophyllotoxin has the strongest activity but high toxicity, and ART is safe but has poorer action activity, and the combination of the two has excellent combined activity and mitigating effect on drug resistance, show good activities on inhibiting the growth of the K562/ADR and overcoming MDR by overexpression of regulatory P-gp proteins, which has a good potential for development.

Shareef et al. [36] introduced aromatic acids, alkynoic acids and acrylic acid derivatives to form a series of target products using the classical esterification reaction system. Among them, compound 8 (Fig. 6) was the most active against A549 cells, and inhibited AKT expression and affected the cell cycle.

The experimental results also showed that DPMA inhibited the formation of tubular structures in endothelial cells and was antiangiogenic, providing a new idea for podophyllotoxin derivatives modified at the C-4^′^ position. Another class of DPT derivatives was found to inhibit the cell cycle arrest of A-549 in G1 phase by introducing piperazine substituents, different from the mechanism of action of DPT. Oh’s research team [75] found that unmodified podophyllotoxin can occupy an important action site of c-MET and block its signaling, as well as inhibit drug resistant cancer cells more strongly compared to ordinary lung cancer, which is expected to be developed into a new class of targeted drugs by combining with subsequent structure optimization. Acetylated podophyllotoxin derivatives also inhibited A549 and NCI-H1299 [76], and treatment with different doses resulted in decreased levels of Cdc2 and CCNB1, increased levels of p21, cleaved caspase-3/-8/-9, activation of the ER pathway, and induction through multiple pathways of NSCLC cell death.

Despite promising *in vitro* results, current studies on podophyllotoxin derivatives in lung cancer therapy have certain limitations. Most of the evidence comes from cell line models, and there is a lack of comprehensive *in vivo* studies, including pharmacokinetics, tissue distribution. Additionally, although insights into the mechanisms of action have been elucidated, the potential for off-target effects and the risk of developing resistance with prolonged treatment are not fully understood.

### 3.3 Prostate Cancer

Podophyllotoxin itself has inhibitory effects on androgen-independent prostate cancer (PC-3) and androgen-dependent prostate cancer (LNCaP), but it is not directly used in clinical treatment because of its high toxicity [77]. Incorporation of amino acids at the C-4 position enhances the hydrophobicity and selective toxicity of the compounds, and the inhibitory effect on PC-3M cells (IC_50_ = 1.28 nM) was superior to that of the parent (IC_50_ = 234.90 nM), inducing apoptosis through inhibition of the PI3K/Akt signaling pathway [78].

Triazole ring-substituted derivatives also have good activity against prostate cancer [32]. The triazole substitution was followed by the introduction of para-aromatic carboxylic acid esters, which were found to be active in several tumor cell lines, especially against PC-3, and showed superior effects to azithromycin and etoposide action in the multidrug resistant cell line K562/ADR. Introduction of 4-cyanophenyl inhibited the migration and colony forming ability of PC-3 cells [79]. The introduction of aryl isothiocyanates at the same position resulted in good activity against prostate cancer cell line DU-145 and a safety window of up to 81.7-fold for normal prostate cells [80]. Reddy et al. [81] found that derivatives with a substituent containing an aryl group attached to a triazolamide could synergistically exert an inhibitory effect on DU-145 cells through a variety of mechanisms, including inhibition of DNA topoisomerase IIα activity, suggesting a good prospect for the development of triazolium ring-substituted derivatives. It has been reported that DPT can resist the autophagy mechanism of cancer cells by stimulating the mitochondrial production of ROS and activating the ERK signaling pathway, and that inhibition of autophagy enhances the apoptosis-inducing effect of the drug [82]. If autophagy inhibitors such as 3-MA, Hydroxychloroquine, chloroquine, etc., are used in combination at the same time, they can make the exertion of DPT’s more complete effect. However, these studies are mostly limited to *in vitro* experiments; issues like poor water solubility, off-target toxicity, and potential drug resistance remain challenges. Future directions should include *in vivo* efficacy and safety studies and exploration of combination strategies to enhance therapeutic potential and overcome resistance.

### 3.4 Other Cancers

**Figure 7:**
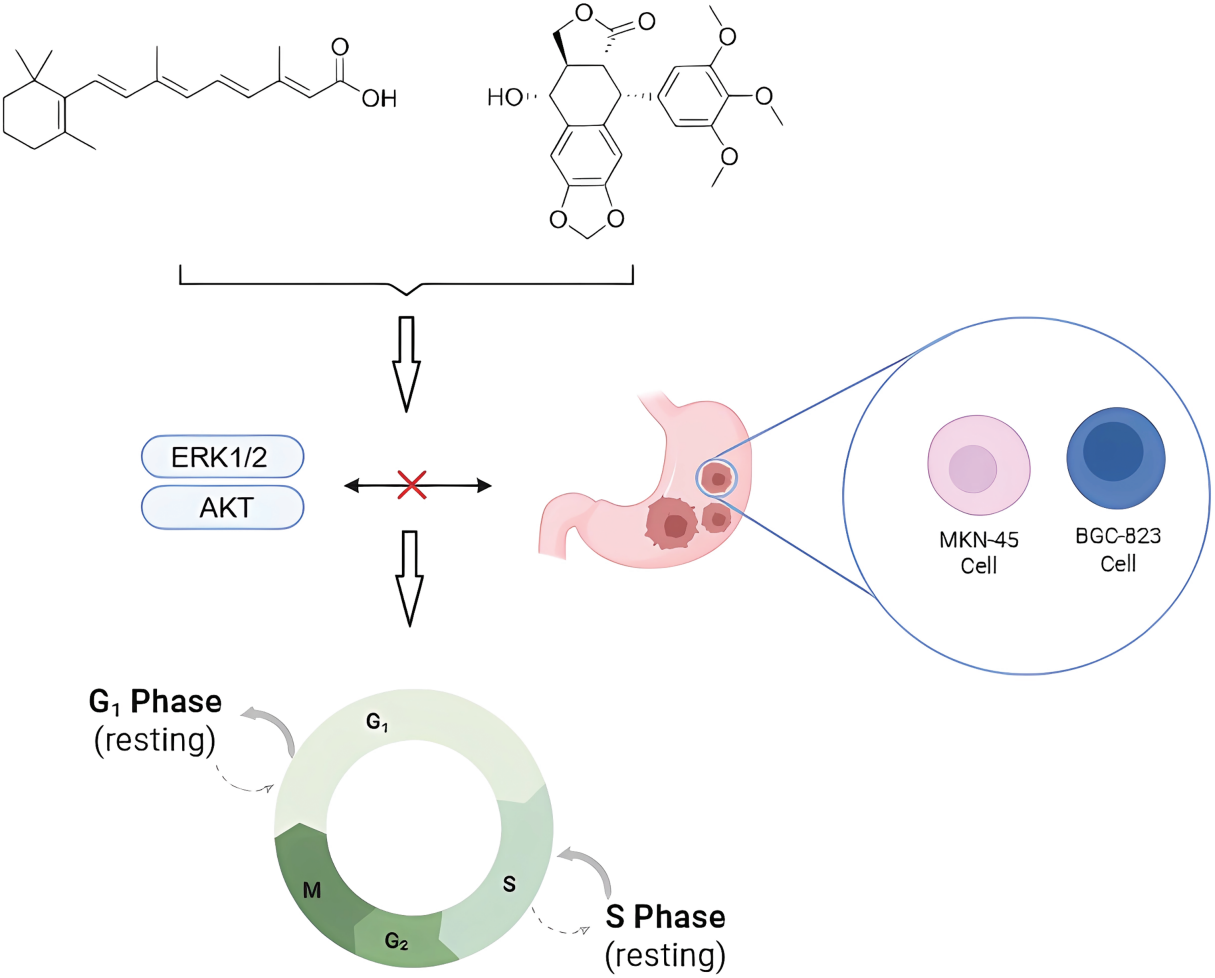
Effects of natural product combinations on tumor cell cycle and signaling pathways in gastric cancer cells. Podophyllotoxin and all-trans retinoic acid interfered with the cycle progression of tumor cells (e.g., MKN-45 and BGC-823 cells) by inhibiting the ERK1/2 and AKT signaling pathways, causing cellular blockade in the G_1_ and S phases, respectively, and thus inhibiting the occurrence and development of cancer. Created in https://BioRender.com

Zhang et al. [83] linked all-trans retinoic acid (ATRA) to PTOX and applied the heterocarbons to gastric cancer cells MKN-45 and BGC-823, showing superior activity to ATRA and etoposide. The results of the biological mechanism showed that the heterodimers caused MKN-45 cells to stop in G1 phase and BGC-823 cells in S phase. It mainly acts by blocking ERK1/2 and AKT signaling in gastric cancer cells (Fig. 7). In addition to the clinically used etoposide and teniposide, various derivatives of PTOX such as Podophyllotoxin-indirubin hybrid (Da-1) [84], GL33157 [85], and TOP5358 [86] show inhibitory effects on leukemia cells *in vitro* [87].

Etoposide is widely used in the treatment of acute myeloid leukemia (AML) and acute lymphoblastic leukemia (ALL) to induce apoptosis of leukemia cells by interfering with DNA replication and cell division. Cao’s research team [88] synthesized the podophyllotoxin derivative CIP-36, which showed a significant inhibitory effect on the adriamycin-resistant sub-strain K562/A02, with good multidrug resistance and low cytotoxicity to normal human vascular endothelial cells and fibroblasts. The results of cleavage assay showed that CIP-36 could inhibit the activity of flutter isomerase IIα and enhance the formation of DNA breakage complexes, and the effect was better than that of etoposide, which may have higher targeting in molecular mechanism. Not only that, CIP-36 also showed the effect of interfering with the cell cycle, interfering with DNA replication and chromosome segregation, and causing the cells to accumulate in large quantities in the G2/M phase, which played an inhibitory role. The natural compound 4^′^-demethyl-deoxypodophyllotoxin glucoside reduces colon cancer cell invasion by activating the Chk2 pathway and inhibiting the Vimentin protein, thereby blocking the epithelial-mesenchymal transition process [86,88]. Linking the novel dendritic macromolecule with podophyllotoxin can improve water solubility and reduce toxicity, and can effectively reduce the expression level of IL-6, attenuate liver histopathological damage, and inhibit the further development of hepatocellular carcinoma [89]. In pancreatic cancer, high expression of K17 predicts cellular resistance to chemotherapeutic agents, and podophyllotoxin inhibits the viability of K17-positive PDAC cells and is effective in reducing tumor volume when combined with gemcitabine [90]. Malignant glioma is the most common primary brain tumor and remains challenging to treat due to its highly invasive nature and resistance to conventional therapies. In this study, deoxypodophyllotoxin (DPT) was shown to reduce the viability of glioma cell lines (C6, SHG-44, and U87) in a dose and time dependent manner, as demonstrated by MTT and colony formation assays [28]. Further analysis revealed that DPT induced non-apoptotic cell death, marked by increased LDH release, mitochondrial depolarization assessed by JC-1 staining, and nuclear translocation of apoptosis-inducing factor (AIF), visualized via confocal microscopy and Western blot. Mechanistically, DPT triggered the hyperactivation of PARP-1 and accumulation of cytoplasmic PAR polymers—hallmarks of parthanatos—which were significantly reduced by PARP-1 knockdown using siRNA or pharmacological inhibition with 3-aminobenzamide. In a glioma xenograft model in nude mice, DPT treatment led to a marked decrease in tumor volume and weight, along with elevated PARP-1 levels, cytoplasmic PAR accumulation, and AIF nuclear translocation in tumor tissues, confirming that parthanatos also occurred *in vivo*. These effects were closely linked to the overproduction of reactive oxygen species (ROS), which was detected through DCFH-DA fluorescence assays and a drop in intracellular glutathione levels; importantly, pretreatment with the antioxidant NAC or PARP-1 inhibitor 3AB suppressed ROS generation, inhibited PARP-1 activation, stabilized mitochondrial function, and improved cell survival. Together, these findings indicate that DPT exerts its antiglioma effect by inducing ROS-mediated parthanatos, highlighting its potential as a therapeutic agent against malignant glioma.

### 3.5 Non-Cancerous Diseases

Podophyllotoxin and its derivatives not only have research potential in the therapeutic field of cancer, but also have good activity in other medical fields such as antiviral [91], antibacterial, antiparasitic [92], antioxidant activity [84,93].

Podophyllotoxin exhibits significant antiviral activity against a variety of clinically relevant viruses. Studies have shown that it inhibits human papillomavirus (HPV), herpes simplex virus type 1 (HSV-1), Measles virus, cytomegalovirus (CMV), and Sindbis virus. Acts by interfering with the expression of virus-associated proteins and the host cell cycle [94]. Sowmya et al. [95] synthesized a series of podophyllotoxin derivatives and tested their antimicrobial activities, the results showed that the compounds containing electron withdrawing groups and branched alkyl substituents exhibited good antimicrobial activities against bacteria and fungi, and this class of derivatives could provide candidates for the development of novel antimicrobial agents to guide drug design. By retaining the key tetralone skeleton of podophyllotoxin and introducing pyrazole, the analogs were found to have decent antibacterial and antifungal activity [96], providing a reference for the design of more potent and less toxic compounds derived from podophyllotoxin. The PTOX extract could completely inhibit the growth of *Bacillus cereus* [97], consistent with the predicted results, but was almost ineffective against Gram negative bacteria, possibly acting by targeting the PhoP/PhoQ system and the Quorum sensing system. Podophyllotoxin derivatives can be made into an ointment to be applied to the affected area to treat genital warts and can be used in combination with imiquimod to reduce the risk of recurrence [98–100]. The main active ingredient in the already marketed Wartec Creme and condylox® is podophyllotoxin.

## 4 Discussion

**Figure 8:**
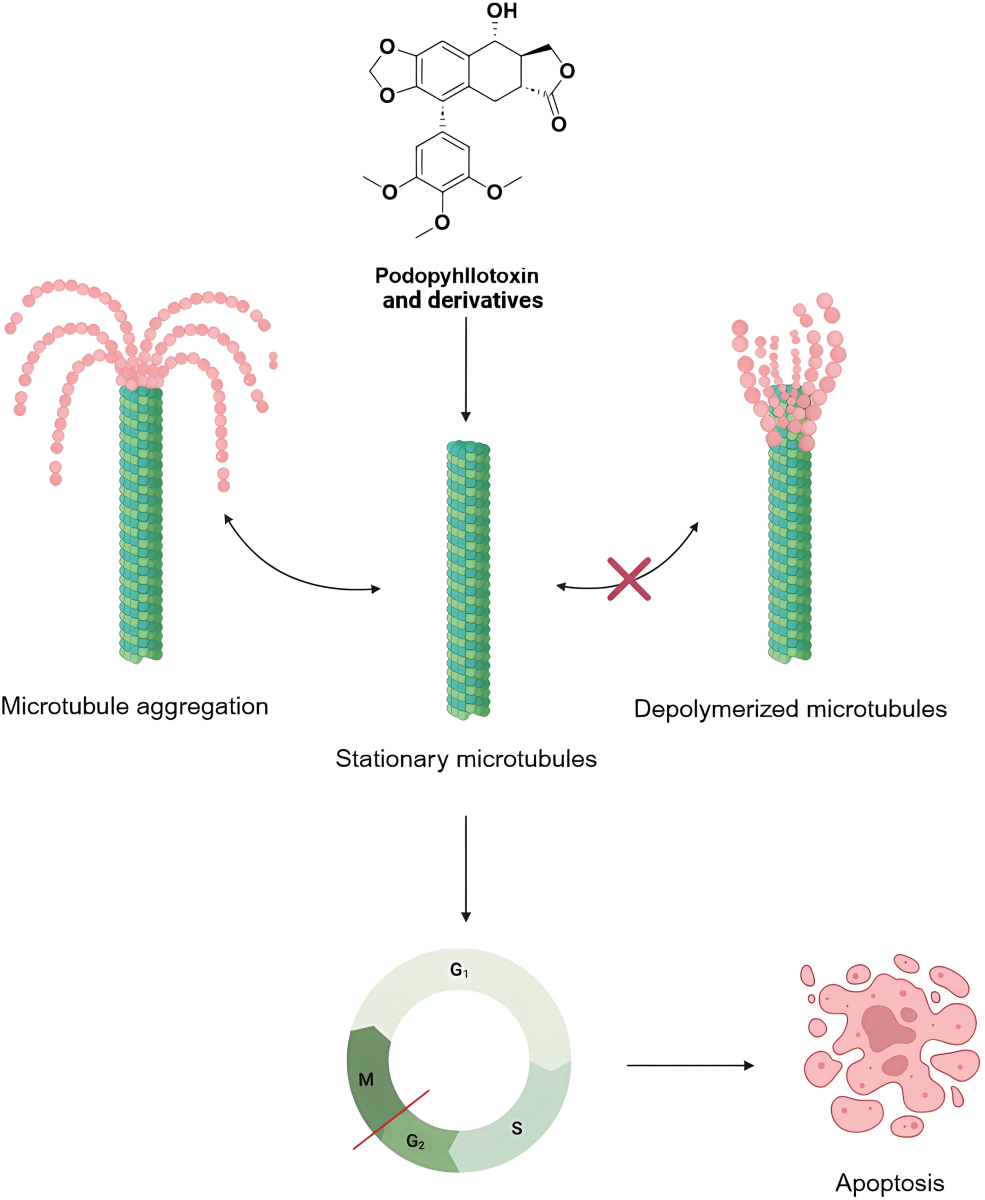
Mechanisms of podophyllotoxin and its derivatives in affecting microtubules and regulating the cell cycle. Podophyllotoxin and its derivatives inhibit microtubule polymerization or stabilize microtubules, keep microtubules in a quiescent state, and block the normal microtubule depolymerization process, leading to cell cycle arrest in G2/M phase, and ultimately inducing apoptosis. Created in https://BioRender.com

Microtubules are formed by the dimerization of α and β microtubule proteins and are widely distributed in the cytoplasm, which play an important role in the formation of cytoskeleton, secretion of vesicles, cell division, and the formation of cilia and flagellum [101]. A large number of longitudinally arranged microtubules can form spindle bodies, which play an important role in mitosis. Currently, anticancer drugs targeting microtubules can be divided into two mechanisms of action: microtubule-destabilizing agents and microtubule-stabilizing agents. Podophyllotoxin is a natural microtubule-destabilizing agents, which can affect the stability of microtubules by inhibiting their polymerization, interfering with the formation of the spindle, and affecting the normal progression of mitosis, so as to make the cell cycle stay in the G2/M phase, the phase of the cell cycle which critically depends on the function of microtubules (Fig. 8). microtubule function in the G2/M phase. Numerous studies have shown that even when the structure of podophyllotoxin is modified, most of the derivatives retain the inhibition of microtubule polymerization, and have demonstrated promising inhibitory effects in *in vitro* experiments in breast, cervical, lung, gastric, and glioblastoma cancers, as well as in colorectal cancers [102]. At the molecular level, podophyllotoxin binds to β-microtubulin through hydrogen bonds, pi-alkyl, and carbon-hydrogen interactions to induce conformational changes. Interferes with the association between microtubulin dimers and prevents the addition of new microtubulin heterodimers to the positive end of the microtubule. Promotes depolymerization by impeding microtubule growth and interferes with the cell cycle by disrupting the dynamic instability necessary for mitotic spindle formation and chromosome alignment. These changes further extend the apoptotic signaling pathway, finally causing the cell death process [11].

The cell cycle is a continuous process of constant cycling, which is regulated by two key molecules, cell cycle proteins and cell cycle protein-dependent kinases (CDKs), and other inhibitory factors, and is divided into four phases, G1, S, G2 and M [103]. The cell cycle is a continuous process of cell proliferation and apoptosis. Abnormal cycle progression may lead to unlimited cell proliferation or apoptosis, which is one of the key targets for tumor cell inhibition. Currently, a variety of targeted drugs act on CDKs to induce cycle arrest and apoptosis [104–106]. Podophyllotoxin and its derivatives can not only destabilize microtubules to arrest cells in G2/M phase, but also regulate the expression of related proteins, such as suppression of cell cycle proteins B1, CDK1, Cyclin E, and Cdc25C, DPT derivatives can inhibit the arrest of lung cancer cells A549 in the G1 phase, exemplifying that altering the structure may result in a different action effect.

Podophyllotoxin can play a key role in tumor cell proliferation, migration and programmed death by modulating several key signaling pathways that regulate cellular life activity. Studies have shown that cancer is regulated by a variety of signaling pathways such as MAPK/ERK [107–109], PI3K/Akt/mTOR [110–112], NF-κB [113], p53/p21 [114,115], Cyclin B1/Cdc2 [116–118] and other signaling networks to inhibit the activity in multiple targets, and depth explorations of the mechanisms of these pathways can better reveal the molecular basis of antitumor effects. Mitogen-activated protein kinase (MAPK), which is mainly composed of ERK1/2, ERK5, p38 and JNK, plays a dual role in cell regulation, either promoting or inhibiting apoptosis [119]. Podophyllotoxin can reduce the phosphorylation level of ERK1 and inhibit cancer cell proliferation. The intrinsic activation pathway of apoptosis is mainly regulated by the dynamics of antiapoptotic and pro-apoptotic proteins. Derivatives containing the structure of podophyllotoxin can enhance the expression of pro-apoptotic proteins Bax/Bcl-2, disrupt the mitochondrial membrane, induce the release of cytochrome c and activate the caspase pathway to play a role. The PI3K/Akt/mTOR pathway is one of the important targets for anticancer drug development. Exogenous growth factors activate PI3K and recruit Akt and PDK1 to the cell membrane, and after phosphorylation of the key Akt sites Thr308 and Ser473, they inhibit pro-apoptotic protein activity and activate mTORC1 to promote cell growth. In cancer cells, PIK3CA activating mutations or deletions are common, and Akt overexpression prevents apoptosis or sustained activation of mTOR, and drug resistance occurs. Podophyllotoxin down-regulates the phosphorylation level of Akt or mTOR to inhibit tumor cell proliferation; meanwhile, it up-regulates PTEN to inhibit Akt activation [120].

## 5 Conclusion

This paper systematically summarizes Podophyllotoxin and its series of commonly active derivatives, covering a wide range of modification methods, conformational relationships and biological activities. Introducing different aromatic heterocycles, amino acids, sugar groups, and fatty acid substituents at key sites of the parent (e.g., C-4) to improve stability and water solubility, targeting selectivity, and to reduce autotoxicity, these modifications not only enhance cytotoxicity, but also potentiate podophyllotoxin inhibitory effects. Blocking the process of microtubule protein polymerization, inhibiting mitosis, arresting the cell cycle in the G2/M phase, and promoting the induction of apoptosis, it exhibits a variety of antitumor activities.

Currently, the activity of podophyllotoxin derivatives remains at the cellular level, and toxicological studies and *in vivo* efficacy evaluations are lacking, so further comprehensive evaluations are needed to promote the translation of potential derivatives to the clinic. Moreover, with the emergence of large-scale genomics databases and algorithms based on artificial intelligence and machine learning provides a valuable platform, podophyllotoxin and its derivatives will have broader indications [121]. Finally, combining podophyllotoxin derivatives with PROTAC technology provides a promising strategy for the development of natural product-based targeted protein degraders. This approach may open new opportunities for selective degradation of disease-causing proteins and expand the therapeutic scope of podophyllotoxin-based drugs, especially for cancer therapy [122].

## Data Availability

Not applicable.

## Abbreviations

PTOX: Podophyllotoxin
DPT: Deoxypodophyllotoxin
PI3K/Akt/mTOR: Phosphoinositide 3-kinase/Protein kinase B (Akt)/Mammalian target of rapamycin
NF-κB: Nuclear Factor kappa-light-chain-enhancer of activated B cells
PLK1: Polo-like kinase 1
GMP-AMP: Guanosine monophosphate-adenosine monophosphate
p38 MAPK: p38 Mitogen-Activated Protein Kinase
Bcl-2: B-cell lymphoma 2
cGAS-STING: Cyclic GMP-AMP synthase–stimulator of interferon genes signaling pathway
CDK1: Cyclin-Dependent Kinase 1
ROS: Reactive Oxygen Species
RIPK1: Receptor-Interacting Protein Kinase 1
RIPK3: Receptor-Interacting Protein Kinase 3
MLKL: Mixed Lineage Kinase Domain-Like protein
EMT: Epithelial-Mesenchymal Transition
MMPs: Matrix Metalloproteinases
p53: Protein Tumor Protein p53
IκBα: Inhibitor of kappa B alpha
DAMPs: Damage-Associated Molecular Patterns
PARP-1: Poly (ADP-ribose) Polymerase 1
AIF: Apoptosis-Inducing Factor
PLK1: Polo-Like Kinase 1
CDK1: Cyclin-Dependent Kinase 1
MCF-7: Michigan Cancer Foundation-7
HDAC: Histone Deacetylase
EGFR: Epidermal Growth Factor Receptor
MTT: 3-(4,5-dimethylthiazol-2-yl)-2,5-diphenyltetrazolium bromide
DPMA: 4^′^-Demethylepipodophyllotoxin-piperidinylmethanone derivative
Cdc2: Cell division control protein 2
ER: Endoplasmic Reticulum
ERK: Extracellular signal-Regulated Kinase
Chk2: Checkpoint kinase 2
KRAS: Kirsten Rat Sarcoma Viral Oncogene Homolog
A549: Adenocarcinomic human alveolar basal epithelial cells
H460: NCI-H460 (Non-Small Cell Lung Carcinoma cells)
WI-38: Wistar Institute-38
NCI-H1299: National Cancer Institute-H1299
CCNB1: Cyclin B1
K17: Keratin 17
PDAC: Pancreatic Ductal Adenocarcinoma
LDH: Lactate Dehydrogenase
JC-1: 5,5^′^,6,6^′^-Tetrachloro-1,1^′^,3,3^′^-tetraethylbenzimidazolylcarbocyanine iodide
DCFH-DA: 2^′^,7^′^-Dichlorodihydrofluorescein diacetate
PhoP: Phosphate regulon response regulator PhoP
PhoQ: Phosphate regulon sensor kinase PhoQ

## References

[1] Ardalani H, Avan A, Ghayour-Mobarhan M. Podophyllotoxin: a novel potential natural anticancer agent. Avicenna J Phytomed. 2017;7(4):285–94; 28884079 PMC5580867

[2] Podwissotzki V. On the active constituents of podophyllin. Am J Pharm. 1882;102:44–50.

[3] Kluska M, Woźniak K. Natural polyphenols as modulators of etoposide anti-cancer activity. Int J Mol Sci. 2021;22(12):6602. doi:10.3390/ijms22126602; 34202987 PMC8235666

[4] Szlachta K, Manukyan A, Raimer HM, Singh S, Salamon A, Guo W, et al. Topoisomerase II contributes to DNA secondary structure-mediated double-stranded breaks. Nucleic Acids Res. 2020;48(12):6654–71. doi:10.1093/nar/gkaa483; 32501506 PMC7337936

[5] Leng J, Zhao Y, Sheng P, Xia Y, Chen T, Zhao S, et al. Discovery of novel N-heterocyclic-fused deoxypodophyllotoxin analogues as tubulin polymerization inhibitors targeting the colchicine-binding site for cancer treatment. J Med Chem. 2022;65(24):16774–800. doi:10.1021/acs.jmedchem.2c01595; 36471625

[6] Ravelli RBG, Gigant B, Curmi PA, Jourdain I, Lachkar S, Sobel A, et al. Insight into tubulin regulation from a complex with colchicine and a stathmin-like domain. Nature. 2004;428(6979):198–202. doi:10.1038/nature02393; 15014504

[7] Seo JH, Yoon G, Park S, Shim JH, Chae JI, Jeon YJ. Deoxypodophyllotoxin induces ROS-mediated apoptosis by modulating the PI3K/AKT and p38 MAPK-dependent signaling in oral squamous cell carcinoma. J Microbiol Biotechnol. 2022;32(9):1103–9. doi:10.4014/jmb.2207.07012; 36039387 PMC9628964

[8] Lee SO, Joo SH, Kwak AW, Lee MH, Seo JH, Cho SS, et al. Podophyllotoxin induces ROS-mediated apoptosis and cell cycle arrest in human colorectal cancer cells via p38 MAPK signaling. Biomol Ther. 2021;29(6):658–66. doi:10.4062/biomolther.2021.143; 34642263 PMC8551740

[9] Shi MX, Ding X, Tang L, Cao WJ, Su B, Zhang J. PROTAC EZH2 degrader-1 overcomes the resistance of podophyllotoxin derivatives in refractory small cell lung cancer with leptomeningeal metastasis. BMC Cancer. 2024;24(1):504. doi:10.1186/s12885-024-12244-3; 38644473 PMC11034131

[10] Han J, Hu S, Hu Y, Xu Y, Hou Y, Yang Y, et al. Discovery of podofilox as a potent cGAMP-STING signaling enhancer with antitumor activity. Cancer Immunol Res. 2023;11(5):583–99. doi:10.1158/2326-6066.CIR-22-0483; 36921097

[11] Guo Y, Chen B, Guo J, Jiang P, Wang J, Sun W. Design, synthesis and biological evaluation of novel podophyllotoxin derivatives as tubulin-targeting anticancer agents. Pharm Biol. 2024;62(1):233–49. doi:10.1080/13880209.2024.2318350; 38393642 PMC10896134

[12] Bray F, Laversanne M, Sung H, Ferlay J, Siegel RL, Soerjomataram I, et al. Global cancer statistics 2022: globocan estimates of incidence and mortality worldwide for 36 cancers in 185 countries. CA Cancer J Clin. 2024;74(3):229–63. doi:10.3322/caac.21834; 38572751

[13] Duan C, Yu M, Xu J, Li BY, Zhao Y, Kankala RK. Overcoming cancer multi-drug resistance (MDR): reasons, mechanisms, nanotherapeutic solutions, and challenges. Biomed Pharmacother. 2023;162:114643. doi:10.1016/j.biopha.2023.114643; 37031496

[14] Nishad S, Hasan SM, Kushwaha SP, Singh K, Kumar A, Suvaiv S. Anticancer structure activity relationship of podophyllotoxin of various species of Podophyllum. Ann Phytomed Int J. 2024;13(1):70–8388. doi:10.54085/ap.2024.13.1.6.

[15] Zhang L, Wang H, Sun X, Wei C, Wang J. Design of podophyllotoxin-based hybrid compounds as potential anticancer agents. Lett Drug Des Discov. 2024;21(11):1895–903. doi:10.2174/1570180820666230606161639.

[16] Xiao J, Gao M, Sun Z, Diao Q, Wang P, Gao F. Recent advances of podophyllotoxin/epipodophyllotoxin hybrids in anticancer activity, mode of action, and structure-activity relationship: an update (2010–2020). Eur J Med Chem. 2020;208(1):112830. doi:10.1016/j.ejmech.2020.112830; 32992133

[17] Liu YQ, Tian J, Qian K, Zhao XB, Morris-Natschke SL, Yang L, et al. Recent progress on C-4-modified podophyllotoxin analogs as potent antitumor agents. Med Res Rev. 2015;35(1):1–62. doi:10.1002/med.21319; 24827545 PMC4337794

[18] Lu B, Xu L, Chen H, Yu G, Khow K, Yang S, et al. C-4 analogues of podophyllotoxin as tubulin inhibitors: synthesis, biological evaluation, and structure-activity relationship. Nat Prod Res. 2024;19(2):1–7. doi:10.1080/14786419.2024.2410410; 39360483

[19] Castro MA, Miguel del Corral JM, Gordaliza M, Gómez-Zurita MA, de la Puente ML, Betancur-Galvis LA, et al. Synthesis, cytotoxicity and antiviral activity of podophyllotoxin analogues modified in the E-ring. Eur J Med Chem. 2003;38(10):899–911. doi:10.1016/j.ejmech.2003.05.001; 14575937

[20] Wei J, Chen J, Ju P, Ma L, Chen L, Ma W, et al. Synthesis and biological evaluation of 4β-N-acetylamino substituted podophyllotoxin derivatives as novel anticancer agents. Front Chem. 2019;7:253. doi:10.3389/fchem.2019.00253; 31106192 PMC6491884

[21] Shen S, Tong Y, Luo Y, Huang L, Gao W. Biosynthesis, total synthesis, and pharmacological activities of aryltetralin-type lignan podophyllotoxin and its derivatives. Nat Prod Rep. 2022;39(9):1856–75. doi:10.1039/d2np00028h; 35913409

[22] Jojić AA, Liga S, Uţu D, Ruse G, Suciu L, Motoc A, et al. Beyond essential oils: diterpenes, lignans, and biflavonoids from *Juniperus communis* L. as a source of multi-target lead compounds. Plants. 2024;13(22):3233. doi:10.3390/plants13223233; 39599442 PMC11598787

[23] Mottaghi S, Abbaszadeh H. A comprehensive insight into the antineoplastic activities and molecular mechanisms of deoxypodophyllotoxin: recent trends, challenges, and future outlook. Eur J Pharmacol. 2022;928:175089. doi:10.1016/j.ejphar.2022.175089; 35688183

[24] Zhang T, Wang Y, Inuzuka H, Wei W. Necroptosis pathways in tumorigenesis. Semin Cancer Biol. 2022;86(Pt 3):32–40. doi:10.1016/j.semcancer.2022.07.007; 35908574 PMC11010659

[25] Shi SN, Qin X, Wang HY, Cai ZY. Research progress in necroptosis. Prog Biochem Biophys. 2020;47(7):561–73. doi:10.16476/j.pibb.2019.0209.

[26] Liu ZG, Jiao D. Necroptosis, tumor necrosis and tumorigenesis. Cell Stress. 2019;4(1):1–8. doi:10.15698/cst2020.01.208; 31922095 PMC6946014

[27] Gupta G, Afzal M, Moglad E, Goyal A, Almalki WH, Goyal K, et al. Parthanatos and apoptosis: unraveling their roles in cancer cell death and therapy resistance. EXCLI J. 2025;24:351–80. doi:10.17179/excli2025-8251; 40166425 PMC11956527

[28] Ma D, Lu B, Feng C, Wang C, Wang Y, Luo T, et al. Deoxypodophyllotoxin triggers parthanatos in glioma cells via induction of excessive ROS. Cancer Lett. 2016;371(2):194–204. doi:10.1016/j.canlet.2015.11.044; 26683770

[29] Wang XZ, Liang SP, Chen X, Wang ZC, Li C, Feng CS, et al. TAX1BP1 contributes to deoxypodophyllotoxin-induced glioma cell parthanatos via inducing nuclear translocation of AIF by activation of mitochondrial respiratory chain complex I. Acta Pharmacol Sin. 2023;44(9):1906–19. doi:10.1038/s41401-023-01091-w; 37186123 PMC10462642

[30] Reddy DM, Srinivas J, Chashoo G, Saxena AK, Sampath Kumar HM. 4β-[(4-Alkyl)-1, 2, 3-triazol-1-yl] podophyllotoxins as anticancer compounds: design, synthesis and biological evaluation. Eur J Med Chem. 2011;46(6):1983–91. doi:10.1016/j.ejmech.2011.02.016; 21477899

[31] Zi CT, Li GT, Li Y, Zhou J, Ding ZT, Jiang ZH, et al. Synthesis and anticancer activity of 4β-triazole-podophyllotoxin glycosides. Nat Prod Bioprospect. 2015;5(2):83–90. doi:10.1007/s13659-015-0057-3; 25869591 PMC4402586

[32] Hou W, Zhang G, Luo Z, Su L, Xu H. Click chemistry-based synthesis and cytotoxic activity evaluation of 4α-triazole acetate podophyllotoxin derivatives. Chem Biol Drug Des. 2019;93(4):473–83. doi:10.1111/cbdd.13436; 30394007

[33] Bkhaitan M, Bardaweel SK, Abushaikha G, Mirza AZ, Sweidan KA. Synthesis and antiproliferative activity of 4β-O-substituted, 4β-N-substituted deoxypodophyllotoxin derivatives, and 4β-OH-4^′^-O-substituted podophyllotoxin. ChemistrySelect. 2020;5(47):14924–9. doi:10.1002/slct.202003810.

[34] Paidakula S, Nerella S, Vadde R, Kamal A, Kankala S. Design and synthesis of 4β-Acetamidobenzofuranone-podophyllotoxin hybrids and their anti-cancer evaluation. Bioorg Med Chem Lett. 2019;29(16):2153–6. doi:10.1016/j.bmcl.2019.06.060; 31281022

[35] Sang CY, Tian HZ, Chen Y, Liu JF, Chen SW, Hui L. Synthesis and biological evaluation of 4β-(thiazol-2-yl)amino-4^′^-O-demethyl-4-deoxypodophyllotoxins as topoisomerase-II inhibitors. Bioorg Med Chem Lett. 2018;28(2):71–6. doi:10.1016/j.bmcl.2017.12.012; 29248296

[36] Shareef MA, Duscharla D, Ramasatyaveni G, Dhoke NR, Das A, Ummanni R, et al. Investigation of podophyllotoxin esters as potential anticancer agents: synthesis, biological studies and tubulin inhibition properties. Eur J Med Chem. 2015;89:128–37. doi:10.1016/j.ejmech.2014.10.050; 25462233

[37] Zhang L, Chen F, Zhang Z, Chen Y, Wang J. Synthesis and biological evaluation of a novel artesunate-podophyllotoxin conjugate as anticancer agent. Bioorg Med Chem Lett. 2016;26(1):38–42. doi:10.1016/j.bmcl.2015.11.042; 26615886

[38] Jin Y, Liu J, Huang WT, Chen SW, Hui L. Synthesis and biological evaluation of derivatives of 4-deoxypodophyllotoxin as antitumor agents. Eur J Med Chem. 2011;46(9):4056–61. doi:10.1016/j.ejmech.2011.06.004; 21733601

[39] Zhang X, Rakesh KP, Shantharam CS, Manukumar HM, Asiri AM, Marwani HM, et al. Podophyllotoxin derivatives as an excellent anticancer aspirant for future chemotherapy: a key current imminent needs. Bioorg Med Chem. 2018;26(2):340–55. doi:10.1016/j.bmc.2017.11.026; 29269253

[40] Guo Q, Jiang E. Recent advances in the application of podophyllotoxin derivatives to fight against multidrug-resistant cancer cells. Curr Top Med Chem. 2021;21(19):1712–24. doi:10.2174/1568026621666210113163327; 33441065

[41] Kamal A, Hussaini SM, Malik MS. Recent developments towards podophyllotoxin congeners as potential apoptosis inducers. Anticancer Agents Med Chem. 2015;15(5):565–74. doi:10.2174/1871520614666141130125623; 25469512

[42] Study of etoposide monotherapy in treating patients with recurrent or metastatic breast cancer [Internet]. [cited 2025 Jul 1]. Available from: https://clinicaltrials.gov/study/NCT01492556.

[43] Liu J, He M, Jiang M, Zhou S, Zhang M, Li Y, et al. Pyrotinib combined with metronomic etoposide in heavily pretreated HER2-positive metastatic breast cancer: a single-arm, phase II study. BMC Cancer. 2024;24(1):1290. doi:10.1186/s12885-024-13041-8; 39425028 PMC11487706

[44] Okamoto H, Watanabe K, Kunikane H, Yokoyama A, Kudoh S, Asakawa T, et al. Randomised phase III trial of carboplatin plus etoposide vs split doses of cisplatin plus etoposide in elderly or poor-risk patients with extensive disease small-cell lung cancer: JCOG 9702. Br J Cancer. 2007;97(2):162–9. doi:10.1038/sj.bjc.6603810; 17579629 PMC2360311

[45] Matsumoto M, Minegishi Y, Higa K, Fukuizumi A, Onda N, Takeuchi S, et al. Carboplatin in combination with etoposide for advanced small cell lung cancer complicated with idiopathic interstitial pneumonia: a single-arm phase II study. BMC Pulm Med. 2025;25(1):9. doi:10.1186/s12890-024-03459-y; 39780119 PMC11707951

[46] Owonikoko TK, Dahlberg SE, Sica GL, Wagner LI, Wade JL III, Srkalovic G, et al. Randomized phase II trial of cisplatin and etoposide in combination with veliparib or placebo for extensive-stage small-cell lung cancer: ECOG-ACRIN, 2511 study. J Clin Oncol. 2019;37(3):222–9. doi:10.1200/JCO.18.00264; 30523756 PMC6338394

[47] Chen Z, Chen J, Huang D, Zhang W, Wu L, Yi T, et al. A multicenter, randomized, double-blind, placebo-controlled phase 3 study of Socazolimab or placebo combined with carboplatin and etoposide in the first-line treatment of extensive-stage small cell lung cancer. Signal Transduct Target Ther. 2025;10(1):28. doi:10.1038/s41392-024-02115-5; 39800716 PMC11725569

[48] Liu Y, Huang Q, Tian L, Zheng X, Chen G, Xu H, et al. Neoadjuvant adebrelimab combined with chemotherapy (cisplatin/carboplatin and etoposide) for limited-stage small-cell lung cancer: a study protocol of phase 2 trial (NIUS). BMJ Open. 2025;15(1):e087302. doi:10.1136/bmjopen-2024-087302; 39800398 PMC11752003

[49] Durvalumab ± tremelimumab in combination with platinum based chemotherapy in untreated extensive-stage small cell lung cancer (CASPIAN) (CASPIAN) [Internet]. [cited 2025 Jul 1]. Available from: https://clinicaltrials.gov/study/NCT03043872.

[50] A study evaluating the safety of abt-263 in combination with etoposide/cisplatin in subjects with cancer [Internet]. [cited 2025 Jul 1]. Available from: https://clinicaltrials.gov/study/NCT00878449.

[51] Sacristán JA, Kennedy-Martin T, Rosell R, Cardenal F, Antón A, Lomas M, et al. Economic evaluation in a randomized phase III clinical trial comparing gemcitabine/cisplatin and etoposide/cisplatin in non-small cell lung cancer. Lung Cancer. 2000;28(2):97–107. doi:10.1016/s0169-5002(99)00120-8; 10717327

[52] Gera K, Cline C, Al-Mansour Z, Medvec A, Lee JH, Galochkina Z, et al. A phase ib clinical trial of oral ciprofloxacin and etoposide in subjects with resistant acute myeloid leukemia. Leuk Lymphoma. 2024;65(10):1502–10. doi:10.1080/10428194.2024.2361111; 38841781

[53] Intensified conditioning regimen with high-dose-etoposide for allogeneic hematopoietic stem cell transplantation for adult acute lymphoblastic leukemia [Internet]. [cited 2025 Jul 1]. Available from: https://clinicaltrials.gov/study/NCT01457040.

[54] Dottino PR, Goodman HM, Kredentser D, Rosenberg M, Cohen CJ. Clinical trial of etoposide and cisplatin as salvage therapy in advanced ovarian carcinoma. Gynecol Oncol. 1987;27(3):350–6. doi:10.1016/0090-8258(87)90257-5; 3305186

[55] Etoposide/cisplatin compared with irinotecan/cisplatin for advanced gastrointestinal neuroendocrine tumor G3 type [Internet]. [cited 2025 Jul 1]. Available from: https://clinicaltrials.gov/study/NCT03963193.

[56] Valproate and etoposide for patients with neuronal tumors and brain metastases [Internet]. [cited 2025 Jul 1]. Available from: https://clinicaltrials.gov/study/NCT00513162.

[57] Gaspar N, Hung GY, Strauss SJ, Campbell-Hewson Q, Dela Cruz FS, Glade Bender JL, et al. Lenvatinib plus ifosfamide and etoposide in children and young adults with relapsed osteosarcoma: a phase 2 randomized clinical trial. JAMA Oncol. 2024;10(12):1645–53. doi:10.1001/jamaoncol.2024.4381; 39418029 PMC11581622

[58] Barzaman K, Karami J, Zarei Z, Hosseinzadeh A, Kazemi MH, Moradi-Kalbolandi S, et al. Breast cancer: biology, biomarkers, and treatments. Int Immunopharmacol. 2020;84(1):106535. doi:10.1016/j.intimp.2020.106535; 32361569

[59] Khan MM, Yalamarty SSK, Rajmalani BA, Filipczak N, Torchilin VP. Recent strategies to overcome breast cancer resistance. Crit Rev Oncol Hematol. 2024;197(9036):104351. doi:10.1016/j.critrevonc.2024.104351; 38615873

[60] Hu D, Li Z, Zheng B, Lin X, Pan Y, Gong P, et al. Cancer-associated fibroblasts in breast cancer: challenges and opportunities. Cancer Commun. 2022;42(5):401–34. doi:10.1002/cac2.12291; 35481621 PMC9118050

[61] Zhang W, Liu C, Li J, Liu R, Zhuang J, Feng F, et al. Target analysis and mechanism of podophyllotoxin in the treatment of triple-negative breast cancer. Front Pharmacol. 2020;11:1211. doi:10.3389/fphar.2020.01211; 32848800 PMC7427588

[62] Bozorov K, Zhao J. Aisa HA. 1, 2, 3-Triazole-containing hybrids as leads in medicinal chemistry: a recent overview. Bioorg Med Chem. 2019;27(16):3511–31. doi:10.1016/j.bmc.2019.07.005; 31300317 PMC7185471

[63] Chen H, Zuo S, Wang X, Tang X, Zhao M, Lu Y, et al. Synthesis of 4β-triazole-podophyllotoxin derivatives by azide-alkyne cycloaddition and biological evaluation as potential antitumor agents. Eur J Med Chem. 2011;46(9):4709–14. doi:10.1016/j.ejmech.2011.07.024; 21821321

[64] Zi CT, Xu FQ, Li GT, Li Y, Ding ZT, Zhou J, et al. Synthesis and anticancer activity of glucosylated podophyllotoxin derivatives linked via 4β-triazole rings. Molecules. 2013;18(11):13992–4012. doi:10.3390/molecules181113992; 24232736 PMC6270044

[65] Khan H, Saeedi M, Nabavi SM, Mubarak MS, Bishayee A. Glycosides from medicinal plants as potential anticancer agents: emerging trends towards future drugs. Curr Med Chem. 2019;26(13):2389–406. doi:10.2174/0929867325666180403145137; 29611474

[66] Khan H, Amin S, Tewari D, Nabavi SM, Atanasov AG. Plant-derived glycosides with α-glucosidase inhibitory activity: current standing and future prospects. Endocr Metab Immune Disord Drug Targets. 2019;19(4):391–401. doi:10.2174/1871530319666181128104831; 30484413

[67] Zi CT, Yang L, Zhang BL, Li Y, Ding ZT, Jiang ZH, et al. Synthesis and cytotoxicities of novel podophyllotoxin xyloside derivatives. Nat Prod Commun. 2019;14(7):1934578X19860668. doi:10.1177/1934578x19860668.

[68] Zi CT, Yang L, Kong QH, Li HM, Yang XZ, Ding ZT, et al. Glucoside derivatives of podophyllotoxin: synthesis, physicochemical properties, and cytotoxicity. Drug Des Devel Ther. 2019;13:3683–92. doi:10.2147/DDDT.S215895; 31695335 PMC6815755

[69] Wu GR, Xu B, Yang YQ, Zhang XY, Fang K, Ma T, et al. Synthesis and biological evaluation of podophyllotoxin derivatives as selective antitumor agents. Eur J Med Chem. 2018;155:183–96. doi:10.1016/j.ejmech.2018.05.052; 29886322

[70] Kamal A, Tamboli JR, Ramaiah MJ, Adil SF, Pushpavalli SL, Ganesh R, et al. Quinazolino linked 4β-amidopodophyllotoxin conjugates regulate angiogenic pathway and control breast cancer cell proliferation. Bioorg Med Chem. 2013;21(21):6414–26. doi:10.1016/j.bmc.2013.08.051; 24055291

[71] Benzina S, Harquail J, Jean S, Beauregard AP, Colquhoun CD, Carroll M, et al. Deoxypodophyllotoxin isolated from *Juniperus communis* induces apoptosis in breast cancer cells. Anticancer Agents Med Chem. 2015;15(1):79–88. doi:10.2174/1871520614666140608150448; 24913660

[72] Kratzer TB, Bandi P, Freedman ND, Smith RA, Travis WD, Jemal A, et al. Lung cancer statistics. 2023 Cancer. 2024;130(8):1330–48. doi:10.1002/cncr.35128; 38279776

[73] Le TT, Wu M, Lee JH, Bhatt N, Inman JT, Berger JM, et al. Etoposide promotes DNA loop trapping and barrier formation by topoisomerase II. Nat Chem Biol. 2023;19(5):641–50. doi:10.1038/s41589-022-01235-9; 36717711 PMC10154222

[74] Javidnia K, Miri R, Rezai H, Jafari A, Azarmehr A, Amirghofran Z. Biological activity and aryltetraline lignans of *Linum persicum*. Pharm Biol. 2005;43(6):547–50. doi:10.1080/13880200500220854.

[75] Oh HN, Kwak AW, Lee MH, Kim E, Yoon G, Cho SS, et al. Targeted inhibition of c-MET by podophyllotoxin promotes caspase-dependent apoptosis and suppresses cell growth in gefitinib-resistant non-small cell lung cancer cells. Phytomedicine. 2021;80:153355. doi:10.1016/j.phymed.2020.153355; 33039730

[76] Choi JY, Hong WG, Cho JH, Kim EM, Kim J, Jung CH, et al. Podophyllotoxin acetate triggers anticancer effects against non-small cell lung cancer cells by promoting cell death via cell cycle arrest, ER stress and autophagy. Int J Oncol. 2015;47(4):1257–65. doi:10.3892/ijo.2015.3123; 26314270 PMC4583522

[77] Jiang RW, Zhou JR, Hon PM, Li SL, Zhou Y, Li LL, et al. Lignans from *Dysosma versipellis* with inhibitory effects on prostate cancer cell lines. J Nat Prod. 2007;70(2):283–6. doi:10.1021/np060430o; 17256902 PMC9633130

[78] Zhao Y, Li D, Wei M, Du R, Yan Z. The ester derivatives obtained by C-ring modification of podophyllotoxin induce apoptosis and inhibited proliferation in PC-3M cells via down-regulation of PI3K/Akt signaling pathway. Bioorg Med Chem Lett. 2021;46(5):128174. doi:10.1016/j.bmcl.2021.128174; 34098082

[79] Zilla MK, Nayak D, Vishwakarma RA, Sharma PR, Goswami A, Ali A. A convergent synthesis of alkyne-azide cycloaddition derivatives of 4-α,β-2-propyne podophyllotoxin depicting potent cytotoxic activity. Eur J Med Chem. 2014;77:47–55. doi:10.1016/j.ejmech.2014.02.030; 24607588

[80] Shankaraiah N, Kumar NP, Amula SB, Nekkanti S, Jeengar MK, Naidu VM, et al. One-pot synthesis of podophyllotoxin-thiourea congeners by employing NH_2_SO_3_H/NaI:anticancer activity, DNA topoisomerase-II inhibition, and apoptosis inducing agents. Bioorg Med Chem Lett. 2015;25(19):4239–44. doi:10.1016/j.bmcl.2015.07.100; 26292628

[81] Reddy VG, Bonam SR, Reddy TS, Akunuri R, Naidu VGM, Nayak VL, et al. 4β-amidotriazole linked podophyllotoxin congeners: dNA topoisomerase-IIα inhibition and potential anticancer agents for prostate cancer. Eur J Med Chem. 2018;144:595–611. doi:10.1016/j.ejmech.2017.12.050; 29289884

[82] Hu S, Zhou Q, Wu WR, Duan YX, Gao ZY, Li YW, et al. Anticancer effect of deoxypodophyllotoxin induces apoptosis of human prostate cancer cells. Oncol Lett. 2016;12(4):2918–23. doi:10.3892/ol.2016.4943; 27698880 PMC5038213

[83] Zhang L, Wang J, Liu L, Zheng C, Wang Y. Synthesis and antiproliferative activity of novel all-trans-retinoic acid-podophyllotoxin conjugate towards human gastric cancer cells. Molecules. 2017;22(4):628. doi:10.3390/molecules22040628; 28420180 PMC6154554

[84] Shah Z, Gohar UF, Jamshed I, Mushtaq A, Mukhtar H, Zia-Ui-Haq M, et al. Podophyllotoxin: history, recent advances and future prospects. Biomolecules. 2021;11(4):603. doi:10.3390/biom11040603; 33921719 PMC8073934

[85] Nagar N, Jat R, Saharan R, Verma S, Sharma D, Bansal K. Podophyllotoxin and their glycosidic derivatives. Pharmacophore. 2011;2(2):87–97.

[86] Fan HY, Zhu ZL, Xian HC, Wang HF, Chen BJ, Tang YJ, et al. Insight into the molecular mechanism of podophyllotoxin derivatives as anticancer drugs. Front Cell Dev Biol. 2021;9:709075. doi:10.3389/fcell.2021.709075; 34447752 PMC8383743

[87] Yin M, Fang Y, Sun X, Xue M, Zhang C, Zhu Z, et al. Synthesis and anticancer activity of podophyllotoxin derivatives with nitrogen-containing heterocycles. Front Chem. 2023;11:1191498. doi:10.3389/fchem.2023.1191498; 37234201 PMC10206303

[88] Cao B, Chen H, Gao Y, Niu C, Zhang Y, Li L. CIP-36, a novel topoisomerase II-targeting agent, induces the apoptosis of multidrug-resistant cancer cells *in vitro*. Int J Mol Med. 2015;35(3):771–6. doi:10.3892/ijmm.2015.2068; 25592869

[89] Sharma S, Mehak M, Chhimwal J, Patial V, Sk UH. Dendrimer-conjugated podophyllotoxin suppresses DENA-induced HCC progression by modulation of inflammatory and fibrogenic factors. Toxicol Res. 2019;8(4):560–7. doi:10.1039/c9tx00103d; 31367338 PMC6621132

[90] Pan CH, Otsuka Y, Sridharan B, Woo M, Leiton CV, Babu S, et al. An unbiased high-throughput drug screen reveals a potential therapeutic vulnerability in the most lethal molecular subtype of pancreatic cancer. Mol Oncol. 2020;14(8):1800–16. doi:10.1002/1878-0261.12743; 32533886 PMC7400780

[91] Gordaliza M, Castro MA, del Corral JM, Feliciano AS. Antitumor properties of podophyllotoxin and related compounds. Curr Pharm Des. 2000;6(18):1811–39. doi:10.2174/1381612003398582; 11102564

[92] Gutiérrez-Gutiérrez F, Romo-Mancillas A, Puebla-Pérez AM, Hernández-Hernández JM, Castillo-Romero A. Identification and molecular characterization of the tubulin-podophyllotoxin-type lignans binding site on *Giardia lamblia*. Chem Biol Drug Des. 2019;94(6):2031–40. doi:10.1111/cbdd.13605; 31436919

[93] Strus P, Sadowski K, Ploch W, Jazdzewska A, Oknianska P, Raniszewska O, et al. The effects of podophyllotoxin derivatives on noncancerous diseases: a systematic review. Int J Mol Sci. 2025;26(3):958. doi:10.3390/ijms26030958; 39940726 PMC11816842

[94] Cui Q, Du R, Liu M, Rong L. Lignans and their derivatives from plants as antivirals. Molecules. 2020;25(1):183. doi:10.3390/molecules25010183; 31906391 PMC6982783

[95] Sowmya PT, Paniraj AS, Lokanatha Rai KM, Sudhir A. Synthesis and biological activity of some new podophyllotoxin bearing pyrimidine moiety. Indian J Heterocycl Chem. 2024;34(3):267. doi:10.59467/ijhc.2024.34.267.

[96] Umesha B, Basavaraju YB, Mahendra C. Synthesis and biological screening of pyrazole moiety containing analogs of podophyllotoxin. Med Chem Res. 2015;24(1):142–51. doi:10.1007/s00044-014-1100-3.

[97] Rocha MP, Campana PRV, Scoaris DO, Almeida VL, Lopes JCD, Shaw JMH, et al. Combined *in vitro* studies and *in silico* target fishing for the evaluation of the biological activities of *Diphylleia cymosa* and *Podophyllum hexandrum*. Molecules. 2018;23(12):3303. doi:10.3390/molecules23123303; 30551576 PMC6321136

[98] Zhu P, Qi RQ, Yang Y, Huo W, Zhang Y, He L, et al. Clinical guideline for the diagnosis and treatment of cutaneous warts. J Evid Based Med. 2022;15(3):284–301. doi:10.1111/jebm.12494; 36117295 PMC9825897

[99] Nicolaidou E, Kanelleas A, Nikolakopoulos S, Bezrodnii G, Nearchou E, Gerodimou M, et al. A short, 8-week course of imiquimod 5% cream versus podophyllotoxin in the treatment of anogenital warts: a retrospective comparative cohort study. Indian J Dermatol Venereol Leprol. 2021;87(5):666–70. doi:10.4103/ijdvl.IJDVL_148_19; 31650979

[100] Gilson R, Nugent D, Bennett K, Doré CJ, Murray ML, Meadows J, et al. Imiquimod versus podophyllotoxin, with and without human papillomavirus vaccine, for anogenital warts: the HIPvac factorial RCT. Health Technol Assess. 2020;24(47):1–86. doi:10.3310/hta24470; 32975189 PMC7548868

[101] Nihongaki Y, Matsubayashi HT, Inoue T. A molecular trap inside microtubules probes luminal access by soluble proteins. Nat Chem Biol. 2021;17(8):888–95. doi:10.1038/s41589-021-00791-w; 33941924 PMC8319117

[102] Lopes D, Maiato H. The tubulin code in mitosis and cancer. Cells. 2020;9(11):2356. doi:10.3390/cells9112356; 33114575 PMC7692294

[103] Zhang M, Zhang L, Hei R, Li X, Cai H, Wu X, et al. CDK inhibitors in cancer therapy, an overview of recent development. Am J Cancer Res. 2021;11(5):1913–35; 34094661 PMC8167670

[104] Juric V, Düssmann H, Lamfers MLM, Prehn JHM, Rehm M, Murphy BM. Transcriptional CDK inhibitors CYC065 and THZ1 induce apoptosis in glioma stem cells derived from recurrent GBM. Cells. 2021;10(5):1182. doi:10.3390/cells10051182; 34066147 PMC8151379

[105] Valk E, Örd M, Faustova I, Loog M. CDK signaling via nonconventional CDK phosphorylation sites. Mol Biol Cell. 2023;34(12):pe5. doi:10.1091/mbc.E22-06-0196; 37906435 PMC10846619

[106] Palmer N, Kaldis P. Less-well known functions of cyclin/CDK complexes. Semin Cell Dev Biol. 2020;107(20):54–62. doi:10.1016/j.semcdb.2020.04.003; 32386818

[107] Sun Y, Liu WZ, Liu T, Feng X, Yang N, Zhou HF. Signaling pathway of MAPK/ERK in cell proliferation, differentiation, migration, senescence and apoptosis. J Recept Signal Transduct Res. 2015;35(6):600–4. doi:10.3109/10799893.2015.1030412; 26096166

[108] Pashirzad M, Khorasanian R, Fard MM, Arjmand MH, Langari H, Khazaei M, et al. The therapeutic potential of MAPK/ERK inhibitors in the treatment of colorectal cancer. Curr Cancer Drug Targets. 2021;21(11):932–43. doi:10.2174/1568009621666211103113339; 34732116

[109] Moon H, Ro SW. MAPK/ERK signaling pathway in hepatocellular carcinoma. Cancers. 2021;13(12):3026. doi:10.3390/cancers13123026; 34204242 PMC8234271

[110] Yu L, Wei J, Liu P. Attacking the PI3K/Akt/mTOR signaling pathway for targeted therapeutic treatment in human cancer. Semin Cancer Biol. 2022;85(1):69–94. doi:10.1016/j.semcancer.2021.06.019; 34175443

[111] Leiphrakpam PD, Are C. PI3K/Akt/mTOR signaling pathway as a target for colorectal cancer treatment. Int J Mol Sci. 2024;25(6):3178. doi:10.3390/ijms25063178; 38542151 PMC10970097

[112] Ediriweera MK, Tennekoon KH, Samarakoon SR. Role of the PI3K/AKT/mTOR signaling pathway in ovarian cancer: biological and therapeutic significance. Semin Cancer Biol. 2019;59:147–60. doi:10.1016/j.semcancer.2019.05.012; 31128298

[113] Poma P. NF-κB and disease. Int J Mol Sci. 2020;21(23):E9181. doi:10.3390/ijms21239181; 33276434 PMC7730361

[114] Engeland K. Cell cycle regulation: p53-p21-RB signaling. Cell Death Differ. 2022;29(5):946–60. doi:10.1038/s41418-022-00988-z; 35361964 PMC9090780

[115] Feng J, Xie L, Lu W, Yu X, Dong H, Ma Y, et al. Hyperactivation of p53 contributes to mitotic catastrophe in podocytes through regulation of the Wee1/CDK1/cyclin B1 axis. Ren Fail. 2024;46(2):2365408. doi:10.1080/0886022X.2024.2365408; 38874119 PMC11182053

[116] Xie B, Wang S, Jiang N, Li JJ. Cyclin B1/CDK1-regulated mitochondrial bioenergetics in cell cycle progression and tumor resistance. Cancer Lett. 2019;443:56–66. doi:10.1016/j.canlet.2018.11.019; 30481564 PMC6759061

[117] Hayward D, Alfonso-Pérez T, Gruneberg U. Orchestration of the spindle assembly checkpoint by CDK1-cyclin B1. FEBS Lett. 2019;593(20):2889–907. doi:10.1002/1873-3468.13591; 31469407

[118] Chen NP, Aretz J, Fässler R. CDK1-cyclin-B1-induced kindlin degradation drives focal adhesion disassembly at mitotic entry. Nat Cell Biol. 2022;24(5):723–36. doi:10.1038/s41556-022-00886-z; 35469017 PMC9106588

[119] Johnson GL, Lapadat R. Mitogen-activated protein kinase pathways mediated by ERK, JNK, and p38 protein kinases. Science. 2002;298(5600):1911–2. doi:10.1126/science.1072682; 12471242

[120] McCubrey JA, Steelman LS, Abrams SL, Lee JT, Chang F, Bertrand FE, et al. Roles of the RAF/MEK/ERK and PI3K/PTEN/AKT pathways in malignant transformation and drug resistance. Adv Enzyme Regul. 2006;46(1):249–79. doi:10.1016/j.advenzreg.2006.01.004; 16854453

[121] Zhang K, Yang X, Wang Y, Yu Y, Huang N, Li G, et al. Artificial intelligence in drug development. Nat Med. 2025;31(1):45–59. doi:10.1038/s41591-024-03434-4; 39833407

[122] Chen C, Feng Y, Zhou C, Liu Z, Tang Z, Zhang Y, et al. Development of natural product-based targeted protein degraders as anticancer agents. Bioorg Chem. 2024;153(2021):107772. doi:10.1016/j.bioorg.2024.107772; 39243739

